# Increased MASH-associated liver cancer in younger demographics

**DOI:** 10.1097/HC9.0000000000000629

**Published:** 2025-01-07

**Authors:** Pojsakorn Danpanichkul, Yanfang Pang, Kanokphong Suparan, Thanida Auttapracha, Supapitch Sirimangklanurak, Abdelrahman M. Attia, Chanattha Thimphitthaya, Michelle Shi Ni Law, Zhenning Yu, Mostafa A. Soliman, Natchaya Polpichai, Chanakarn Kanitthamniyom, Donghee Kim, Mazen Noureddin, Amit G. Singal, Karn Wijarnpreecha, Ju Dong Yang

**Affiliations:** 1 Department of Internal Medicine, Texas Tech University Health Sciences Center, Lubbock, Texas, USA; 2 Affiliated Hospital of Youjiang Medical University for Nationalities, Baise, Guangxi, China; 3 National Immunological Laboratory of Traditional Chinese Medicine, Baise, Guangxi, China; 4 Center for Medical Laboratory Science, Affiliated Hospital of Youjiang Medical University for Nationalities, Baise, Guangxi, China; 5 Department of Microbiology, Faculty of Medicine, Chiang Mai University, Chiang Mai, Thailand; 6 Immunology Unit, Department of Microbiology, Faculty of Medicine, Chiang Mai University, Chiang Mai, Thailand; 7 Faculty of Medicine, Chiang Mai University, Chiang Mai, Thailand; 8 Karsh Division of Gastroenterology and Hepatology, Comprehensive Transplant Center, and Samuel Oschin Comprehensive Cancer Institute, Cedars-Sinai Medical Center, Los Angeles, California, USA; 9 Department of Internal Medicine, University of Texas Southwestern Medical Center, Dallas, Texas, USA; 10 Yong Loo Lin School of Medicine, National University of Singapore, Singapore; 11 Harvard Medical School, Boston, Massachusettes, USA; 12 Department of Internal Medicine, Weiss Memorial Hospital, Chicago, Illinois, USA; 13 Division of Gastroenterology and Hepatology, Stanford University School of Medicine, Stanford, California, USA; 14 Houston Research Institute, Houston Methodist Hospital, Houston, Texas, USA; 15 Division of Digestive and Liver Diseases, University of Texas Southwestern Medical Center, Dallas, Texas, USA; 16 Department of Medicine, Division of Gastroenterology and Hepatology, University of Arizona College of Medicine, Phoenix, Arizona, USA; 17 Department of Internal Medicine, Banner University Medical Center, Phoenix, Arizona, USA; 18 BIO5 Institute, University of Arizona College of Medicine-Phoenix, Phoenix, Arizona, USA

**Keywords:** early-onset cancer, epidemiology, gastrointestinal cancer, metabolic dysfunction–associated steatotic liver disease, nonalcoholic fatty liver disease

## Abstract

**Background:**

The incidence of cancer and the prevalence of metabolic disease and metabolic dysfunction–associated steatotic liver disease is increasing in young adults. However, updated global data on metabolic dysfunction–associated steatohepatitis (MASH)-associated primary liver cancer (PLC) in young adults remains scarce.

**Methods:**

This study analyzed data from the Global Burden of Disease study between 2000 and 2021 to assess the age-standardized incidence, mortality, and disability-adjusted life years rates from MASH-associated PLC in young adults (15–49 y).

**Results:**

In 2021, there were 4300 incidence cases, 3550 deaths, and 179,340 disability-adjusted life years from MASH-associated PLC in young adults. Among various etiologies of PLC in young adults, only MASH-associated PLC had increased incidence rates (annual percent change: +0.26, 95% CI: 0.16%–0.35%), with the Eastern Mediterranean region having the largest observed increase (annual percent change: 1.46%, 95% CI: 1.40%–1.51%). In 2021, MASH-associated PLC in young adults made up 6% (+1% from 2000) incident cases, 6% (+2% from 2000) deaths, and 6% (+2% from 2000) disability-adjusted life years of all PLC in this age group. Over half of the countries exhibited an increase in age-standardized incidence rate from MASH-associated PLC in young adults from 2000 to 2021.

**Conclusions:**

The incidence of MASH-associated PLC in young adults is significantly increasing, signaling likely future increases in PLC incidence among older adults as this cohort ages. This trend necessitates urgent strategies worldwide to mitigate the epidemics of MASH-associated PLC in young adults.

## Graphical abstract

**Figure:**
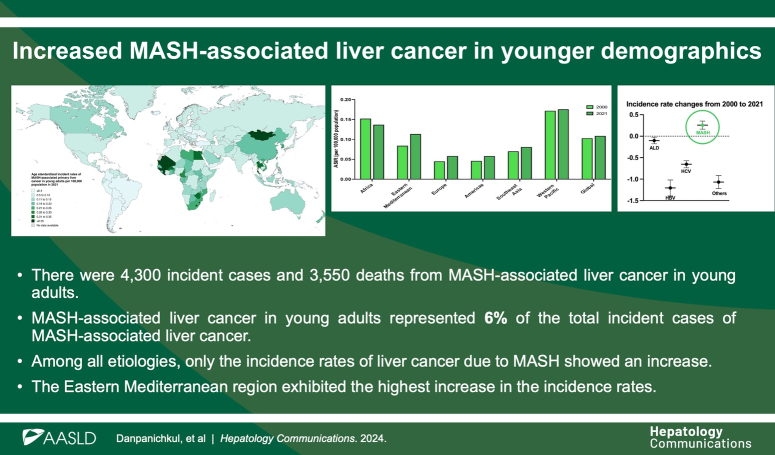

## INTRODUCTION

Cancer is a leading cause of death worldwide, with GLOBOCAN 2020 reporting 19.3 million new cases in that year.^1^ The burden of cancer is not evenly distributed across age groups, with the majority of cases occurring in older adults.^2^ However, there is increasing evidence of a rising incidence of cancer in young adults.^1^^,^^3^^–^^6^ This form of cancer is distinct due to different risk factors, tumor biology, and survivorship compared to cancer in the general population.^2^^,^^7^^,^^8^ Young adults also often face delays in diagnosis for certain cancers, primarily due to the lack of cost-effective early detection methods and the rarity of cancer in this age group.^9^

Primary liver cancer (PLC) is the fourth leading cause of cancer-related death globally.^10^ In 2021, PLC accounted for 483,880 deaths globally.^11^ Unlike many other types of cancer, the incidence of PLC in young adults has decreased in recent decades.^12^^,^^13^ This is likely due to advancements in managing chronic viral hepatitis, including the widespread vaccination against HBV.^14^ However, recent decades have also seen a rising wave of metabolic diseases, particularly metabolic dysfunction–associated steatotic liver disease (MASLD), among young adults.^15^^–^^18^ MASLD is closely linked to various metabolic conditions and significantly increases the risk of progressing to metabolic dysfunction–associated steatohepatitis (MASH) and PLC.^19^ MASH can progress to liver cancer through a series of pathological stages. Initially, lipotoxicity caused by increased endoplasmic reticulum stress, oxidative stress, and activation of the inflammasome leads to hepatocyte injury and cell death. This triggers inflammation and fibrosis, which reactivates molecular pathways and promotes cell proliferation. Over time, these changes alter the liver’s inflammatory environment, depleting protective CD8+ T cells and creating conditions that favor the development of HCC.^20^ Approximately 0.5%–2.6% of individuals with MASH develop PLC.^21^

Given the increased prevalence of metabolic diseases in young adults, coupled with the rising trend of cancer in this age group, these factors may have significantly influenced the recent global epidemiology of MASH-associated PLC in young adults.^1^^,^^17^ Updated data characterizing the burden of MASH-associated PLC at the global, regional, national, sex, and sociodemographic levels is crucial. In this study, we investigated temporal trends in MASH-associated PLC incidence, mortality, and disability-adjusted life years (DALYs) across 204 countries and territories in young adults, using the latest data from the Global Burden of Disease (GBD) Study 2021.^22^

## METHODS

### Data source

The study accessed data on NASH-associated PLC incidence, mortality, and DALYs from 2000 to 2021 using the GBD 2021 data set.^22^ Given the overlap between NASH and MASH data and the recent consensus statement from the American Association for the Study of Liver Diseases, MASH terminology was used instead of NASH.^23^^–^^25^ We evaluated differences in the burden of MASH-associated PLC by sex, region, and country. Data were obtained through the GlobalHealth Data Exchange (GHDx) query tool (http://ghdx.healthdata.org/gbd-results-tool), maintained by the Institute for Health Metrics and Evaluation. This tool provides annual frequencies and age-standardized rates for MASH-associated PLC incidence, deaths, and DALYs by sex, region, and country.

### Estimation methods

The estimation methods for GBD 2021 and the methodology for calculating the burden of MASH-associated PLC were outlined in a previous GBD study and detailed in Supplemental Material S1, http://links.lww.com/HC9/B869.^22^^,^^26^ Standardized coding ensured consistent identification of MASH-associated PLC across various countries and regions. In this study, we defined MASH-associated PLC in young adults as PLC in patients aged 15–49 years. Countries were classified by development level using the sociodemographic index (SDI), which considers total fertility rate, average educational attainment, and income per capita (Supplemental Material S2, http://links.lww.com/HC9/B869). SDI categories are: (1) high (above the 80th percentile), (2) high-middle (60th–79th percentiles), (3) middle (40th–59th percentiles), (4) low-middle (20th–39th percentiles), and (5) low (below the 20th percentile). The burden of MASH-associated PLC was categorized into 6 regions based on the World Health Organization classification: Africa, the Eastern Mediterranean, Europe, the Americas, Southeast Asia, and the Western Pacific. The burden of MASH-associated PLC was also compared to other etiologies, including alcohol-associated liver disease (ALD), chronic hepatitis B, chronic hepatitis C, and other causes.

### Statistical analysis

To provide a comprehensive understanding of the variability in the data and a range of uncertainty in the statistical modeling, each estimate for incidence, deaths, and DALYs in the study was reported with 95% uncertainty intervals (UIs). These intervals are calculated by identifying the 2.5th and 97.5th ranked values across all 1000 draws from the posterior distribution, offering a range that reflects the uncertainty in the statistical modeling. This approach allows for a more thorough understanding of the data variability. Age-standardized rates per 100,000 population were calculated using the GBD 2021 population estimate method, ensuring comparability across different populations and periods.^22^ The study calculated the annual percent change (APC) of age-standardized rates and its 95% CI to assess changes over time. An uptrend is indicated if the APC is positive and the *p* value is <0.05, while a downtrend is indicated if the APC is negative and the *p* value is <0.05. The change is considered insignificant if the *p* value is ≥0.05. This analysis was performed using the Joinpoint regression program, version 4.9.1.0, developed by the Statistical Research and Applications Branch of the National Cancer Institute in Bethesda. We calculated the proportion of young adults with MASH-associated PLC relative to the overall PLC cases in this age group. Further, we stratified MASH-associated PLC cases by specific age brackets within young adults: 15–19, 20–24, 25–29, 30–34, 35–39, 40–44, and 45–49 years. In addition, we compared MASH-associated PLC in young adults with older adults, noting that while the GBD database does not provide a precise age group for individuals over 50 years, it does include data for those over 55 years, which we used as a comparative benchmark.

## RESULTS

### Global burden of MASH-associated PLC in young adults in 2021

**FIGURE 1:**
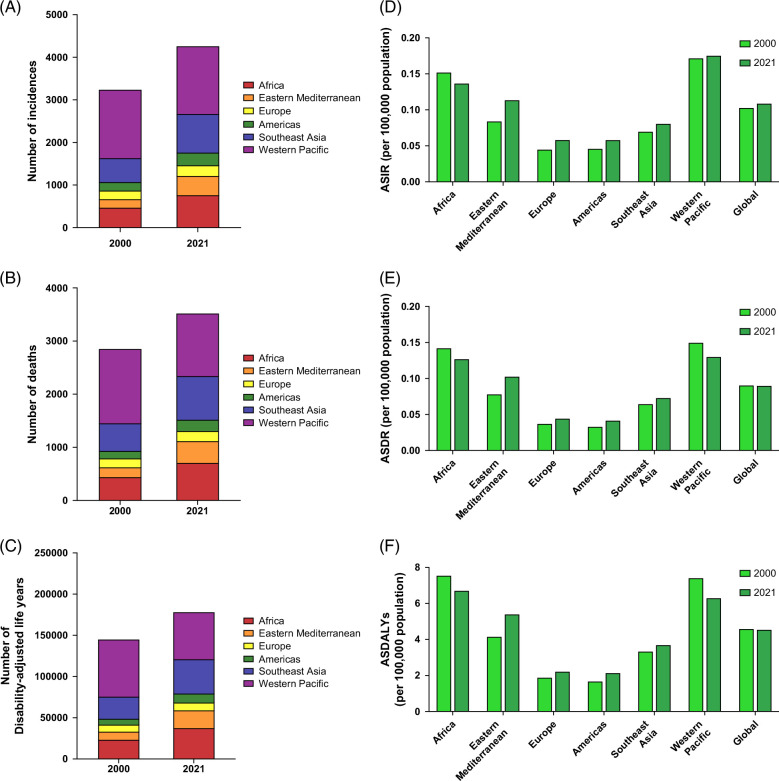
The trend in the burden of MASH-associated primary liver cancer in young adults, stratified by the World Health Organization Region. (A) The number of incidences of MASH-associated primary liver cancer in young adults in 2000 and 2021, stratified by the World Health Organization Region. (B) The number of deaths of MASH-associated primary liver cancer in young adults in 2000 and 2021, stratified by the World Health Organization Region. (C) The number of disability-adjusted life years lost from MASH-associated primary liver cancer in young adults in 2000 and 2021, stratified by the World Health Organization Region. (D) Age-standardized incidence rates of MASH-associated primary liver cancer in young adults in 2000 and 2021 by the World Health Organization Region. (E) Age-standardized death rates for MASH-associated primary liver cancer in young adults in 2000 and 2021 by the World Health Organization Region. (F) Age-standardized disability-adjusted life years MASH-associated primary liver cancer in young adults in 2000 and 2021 by the World Health Organization Region. Abbreviations: ASDALYs, age-standardized disability-adjusted life years; ASDR, age-standardized death rates; ASIR, age-standardized incidence rates; MASH, metabolic dysfunction–associated steatohepatitis.

Globally in 2021, the number of MASH-associated PLC incidences, deaths, and DALYs in young adults was estimated to be 4300 (95% UI: 3310–5510) cases, 3550 (95% UI: 2730–4560) deaths, and 179,340 (95% UI: 140,050–228,420) DALYs (Table 1 and Figures 1A–C). In 2021, age-standardized incidence rate (ASIR), age-standardized death rate (ASDR), and age-standardized DALYs (ASDALYs) were 0.11 (95% UI: 0.08–0.14) per 100,000, 0.09 (95% UI: 0.07–0.12) per 100,000, and 4.54 (95% UI: 3.55–5.78) per 100,000 respectively (Table 1 and Figures 1D–F). ASIR (APC: 0.26%, 95% CI: 0.16%–0.35%) increased, whereas ASDR and ASDALYs remained stable (Table 1).

**TABLE 1:** Incidence, death, disability-adjusted life years, and age-standardized rates of patients with MASH-associated primary liver cancer in young adults in 2021 and changes from 2000 to 2021

	Incidence				Death				Disability-adjusted life years			
	2021 Number (95% UI)	2021 Age-standardized incidence rate (95% UI)	2000– 2021 Annual percent change (95% CI)	*p*	2021 Number (95% UI)	2021 Age-standardized death rate (95% UI)	2000–2021 Annual percent change (95% CI)	*p*	2021 Number (95% UI)	2021 Age-standardized death rate (95% UI)	2000–2021 Annual percent change (95% CI)	*p*
Both	4300 (3310–5510)	0.11 (0.08–0.14)	0.26 (0.16–0.35)	<0.001	3550 (2730–4560)	0.09 (0.07–0.12)	−0.07 (−0.23–0.09)	0.417	179,340 (140,050–228,420)	4.54 (3.55–5.78)	−0.09 (−0.25–0.08)	0.299
By sex												
Female	1920 (1490–2450)	0.1 (0.08–0.13)	0.35 (0.25–0.46)	<0.001	1610 (1260–2060)	0.08 (0.06–0.11)	0.06 (−0.1 to 0.22)	0.485	83,560 (65,770–105,790)	4.29 (3.38–5.43)	0.1 (−0.04 to 0.24)	0.171
Male	2380 (1780–3180)	0.12 (0.09–0.16)	0.18 (0.08–0.27)	<0.001	1940 (1440–2630)	0.1 (0.07–0.13)	−0.17 (−0.34 to 0)	0.047	95,780 (71,860–128,060)	4.79 (3.59–6.4)	−0.2 (−0.37 to −0.02)	0.028
By WHO region												
Africa	770 (520–1120)	0.14 (0.09–0.2)	−0.52 (−0.64 to −0.41)	<0.001	710 (480–1040)	0.13 (0.09–0.19)	−0.55 (−0.69 to −0.42)	<0.001	37,550 (25,480–55,300)	6.71 (4.55–9.88)	−0.58 (−0.71 to −0.45)	<0.001
Eastern Mediterranean	450 (330–610)	0.11 (0.08–0.15)	1.46 (1.4–1.51)	<0.001	410 (290–550)	0.1 (0.07–0.14)	1.28 (1.15–1.4)	<0.001	21,610 (15,690–28,640)	5.4 (3.92–7.16)	1.23 (1.11–1.36)	<0.001
Europe	250 (190–330)	0.06 (0.04–0.08)	1.27 (1.18–1.36)	<0.001	190 (140–250)	0.04 (0.03–0.06)	0.88 (0.47–1.3)	<0.001	9470 (7160–12,330)	2.22 (1.67–2.88)	0.81 (0.42–1.21)	<0.001
Region of the Americas	300 (240–360)	0.06 (0.05–0.07)	1.11 (1.09–1.14)	<0.001	210 (170–260)	0.04 (0.03–0.05)	1.22 (0.89–1.54)	<0.001	10,950 (8780–13,290)	2.14 (1.71–2.59)	1.3 (0.97–1.64)	<0.001
Southeast Asia	910 (690–1140)	0.08 (0.06–0.1)	0.68 (0.63–0.72)	<0.001	820 (630–1030)	0.07 (0.06–0.09)	0.6 (0.5–0.7)	<0.001	41,560 (31,960–51,700)	3.69 (2.84–4.59)	0.48 (0.37–0.59)	<0.001
Western Pacific	1590 (1180–2100)	0.18 (0.13–0.23)	0.06 (−0.18 to 0.3)	0.626	1180 (880–1560)	0.13 (0.1–0.17)	−0.7 (−1.09 to −0.32)	<0.001	57,120 (43,010–75,540)	6.3 (4.74–8.33)	−0.8 (−1.2 to −0.39)	<0.001
By SDI												
Low SDI	550 (360–830)	0.1 (0.07–0.15)	−0.26 (−0.33 to −0.2)	<0.001	510 (330–770)	0.09 (0.06–0.14)	−0.32 (−0.49 to −0.15)	<0.001	27,240 (17,730–41,080)	5.02 (3.27–7.57)	−0.32 (−0.49 to −0.14)	<0.001
Low-middle SDI	970 (720–1250)	0.1 (0.07–0.12)	1.06 (1.01–1.11)	<0.001	890 (670–1150)	0.09 (0.07–0.11)	0.98 (0.91–1.05)	<0.001	46,600 (35,360–59,360)	4.59 (3.48–5.84)	0.91 (0.84–0.98)	<0.001
Middle SDI	1600 (1230–2070)	0.13 (0.1–0.16)	0.46 (0.11–0.82)	0.011	1300 (1010–1690)	0.1 (0.08–0.13)	0.05 (−0.12 to 0.22)	0.55	64,610 (50,180–82,690)	5.15 (4–6.59)	−0.06 (−0.23 to 0.11)	0.5
High-middle SDI	730 (560–950)	0.12 (0.09–0.15)	−0.32 (−0.56 to −0.08)	0.01	560 (420–730)	0.09 (0.07–0.12)	−0.96 (−1.51 to −0.4)	0.001	27,060 (20,580–35,300)	4.3 (3.27–5.61)	−1.06 (−1.45 to −0.66)	<0.001
High SDI	450 (340–580)	0.09 (0.07–0.11)	0.29 (0.22–0.36)	<0.001	280 (210–360)	0.06 (0.04–0.07)	−0.27 (−0.73 to 0.2)	0.257	13,720 (10,450–17,640)	2.73 (2.08–3.51)	−0.16 (−0.63 to 0.3)	0.496

Abbreviations: SDI, sociodemographic index; UI, uncertainty interval; WHO, World Health Organization.

**FIGURE 2:**
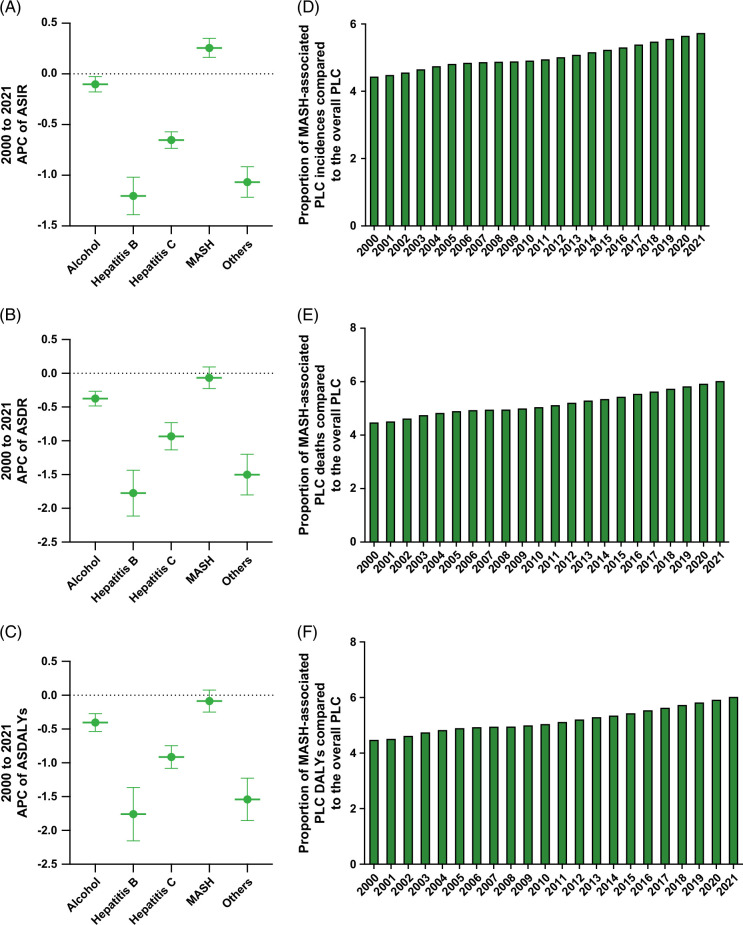
Annual percent change and proportion of MASH-associated primary liver cancer in young adults relative to other etiologies. (A) Annual percent change from 2000 to 2021 of age-standardized incidence rates attributable to primary liver cancer in young adults from MASH and other etiologies. (B) Annual percent change from 2000 to 2021 of age-standardized death rates attributable to primary liver cancer in young adults from MASH and other etiologies. (C) Annual percent change from 2000 to 2021 of age-standardized disability-adjusted life years in young adults attributable to primary liver cancer from MASH and other etiologies. (D) Proportion of incidence of primary liver cancer in young adults from MASH compared to other etiologies from 2000 to 2021. (E) Proportion of death from primary liver cancer in young adults from MASH compared to other etiologies from 2000 to 2021. (F) Proportion of disability-adjusted life years lost from primary liver cancer in young adults from MASH compared to other etiologies from 2000 to 2021. Abbreviations: ALD, alcohol-associated liver disease; APC, annual percent change; ASDALYs, age-standardized disability-adjusted life years; ASDR, age-standardized death rates; ASIR, age-standardized incidence rates; DALY, disability-adjusted life years; MASH, metabolic dysfunction–associated steatohepatitis; PLC, primary liver cancer.

The burden of MASH compared with all etiologies of PLC in young adults is detailed in Supplemental Material S3, http://links.lww.com/HC9/B869 and Supplemental Table S1, http://links.lww.com/HC9/B869. In short, the ASIR increased only in MASH-associated PLC. At the same time, the ASDR and ASDALYs decreased in all etiologies (ALD, HBV, HCC, and other etiologies), except MASH-associated PLC, in which ASDR and ASDALYs remained unchanged. (Figures 2A–C). In 2021, MASH-associated PLC in young adults represented 6% (+1% since 2000) incident cases, 6% (+2% since 2000) deaths, and 6% DALYs (+2% since 2000) of all PLC in young adults (Figures 2D–F).

The burden of MASLD in young adults is designated in Supplemental Table S2, http://links.lww.com/HC9/B869. Briefly, age-standardized prevalence rate, ASDR, and ASDALYs from MASLD were 901.71 (95% UI: 802.31–1012.34), 0.47 (95% UI: 0.32–0.67), and 23.55 (95% UI: 16.25–33.42) per 100,000, respectively. Males have a greater age-standardized prevalence rate, ASDR, and ASDALYs from MASLD than females.

### The burden of MASH-associated PLC in young adults by sex

**FIGURE 3:**
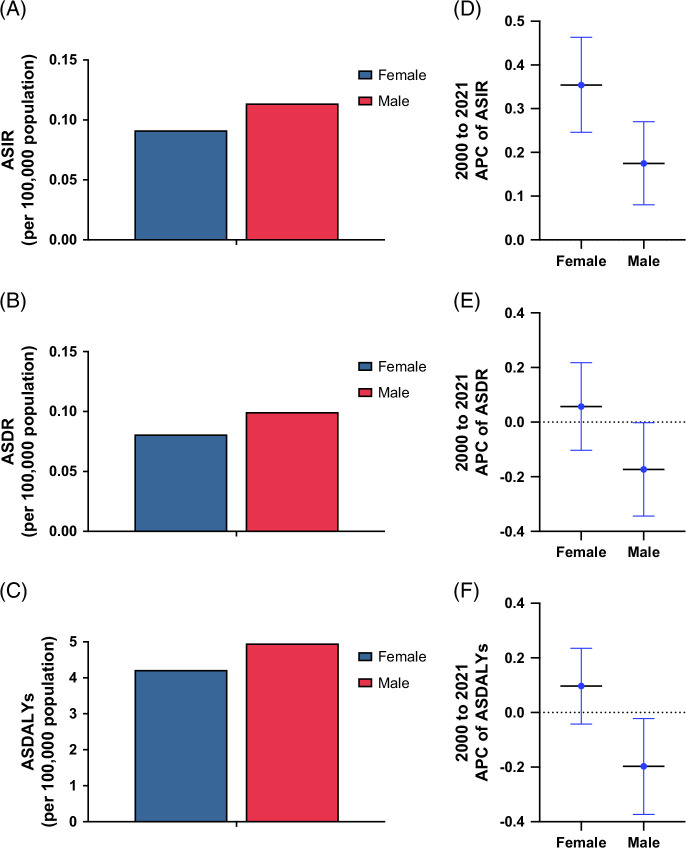
The trend in the burden of MASH-associated primary liver cancer in young adults, stratified by sex. (A) Age-standardized incidence rates of MASH-associated primary liver cancer in young adults in 2021 by sex. (B) Age-standardized death rates for MASH-associated primary liver cancer in young adults in 2021 by sex. (C) Age-standardized disability-adjusted life years MASH-associated primary liver cancer in young adults in 2021 by sex. (D) Annual percent change from 2000 to 2021 of age-standardized incidence rates attributable to MASH-associated primary liver cancer in young adults, by sex. (E) Annual percent change from 2000 to 2021 of age-standardized death rates attributable to MASH-associated primary liver cancer in young adults, by sex. (F) Annual percent change from 2000 to 2021 of age-standardized disability-adjusted life years attributable to MASH-associated primary liver cancer in young adults, by sex. Abbreviations: APC, annual percent change; ASDALYs, age-standardized disability-adjusted life years; ASDR, age-standardized death rates; ASIR, age-standardized incidence rates; MASH, metabolic dysfunction–associated steatohepatitis.

The incidence, death, and DALYs were higher in males compared to females, although the gap has narrowed over time. In 2021, the number of incident MASH-associated PLC cases in young females was 1920 (95% UI: 1490–2450), with 1610 (95% UI: 1260–2060) deaths and 83,560 (95% UI: 65,770–105,790) DALYs. In young males, there were 2380 (95% UI: 1780–3180) incident cases, 1940 (95% UI: 1440–2630) deaths, and 95,780 (95% UI: 71,860–128,060) DALYs (Table 1). ASIR, ASDR, and ASDALYs of young females were 0.10 (95% UI: 0.08–0.13), 0.08 (95% UI: 0.06–0.11), and 4.29 (3.38–5.43) per 100,000 population, respectively. In young males, ASIR, ASDR, and ASDALYs of MASH-associated PLC were 0.12 (95% UI: 0.09–0.16), 0.10 (95% UI: 0.07–0.13), and 4.79 (95% UI: 3.59–6.40) per 100,000 population, respectively. (Figures 3A–C). From 2000 to 2021, ASIR (APC: 0.35%, 95% CI: 0.25%–0.46%) increased, while ASDR and ASDALYs remained stable in females. Meanwhile, in males, ASIR (APC: 0.18%, 95% CI: 0.08%–0.27%) increased, whereas ASDR (APC: −0.17%, 95% CI:−0.34 to 0.00%) and ASDALYs −0.20 (95% CI: −0.37 to −0.02%) decreased (Figures 3D–F).

### The burden of MASH-associated PLC in young adults, by the World Health Organization region

In 2021, the Western Pacific region had the highest MASH-associated PLC incidence (n = 1590), deaths (n = 1180), and DALYs (57120) in young adults (Figures 1A–C). The Western Pacific region exhibited the highest ASIR with a value of 0.18 (95% UI: 0.13–0.23). In comparison, ASDR and ASDALYs were highest in Africa with values of 0.13 (95% UI: 0.09–0.19) and 6.71 (95% UI: 4.55–9.88) per 100,000 population, respectively (Figures 1D–F). Between 2000 and 2021, the Eastern Mediterranean region experienced the highest increase in ASIR (APC: 1.46, 95% CI: 1.40%–1.51%) and ASDR (APC: 1.28%, 95% CI: 1.15%–1.40%), while the Americas had the highest increase in ASDALYs (APC: 1.30%, 95% CI: 0.97%–1.64%) of MASH-associated PLC (Table 1).

### The burden of MASH-associated PLC in young adults, by SDI

**FIGURE 4:**
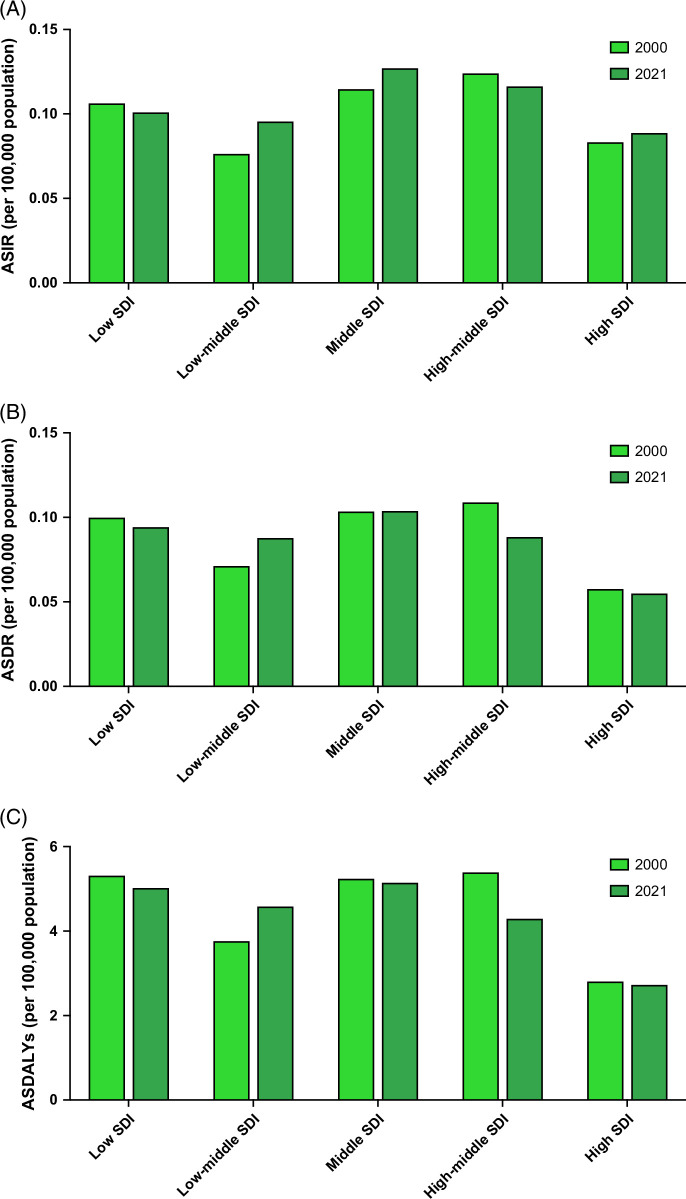
The trend in the burden of MASH-associated primary liver cancer in young adults, stratified by the sociodemographic index. (A) Age-standardized incidence rates attributable to MASH-associated primary liver cancer in young adults in 2000 and 2021 by sociodemographic index. (B) Age-standardized death rates attributable to MASH-associated primary liver cancer in young adults in 2000 and 2021 by the sociodemographic index. (C) Age-standardized disability-adjusted life years attributable to MASH-associated primary liver cancer in young adults in 2000 and 2021 by sociodemographic index. Abbreviations: ASDALYs, age-standardized disability-adjusted life years; ASDR, age-standardized death rates; ASIR, age-standardized incidence rates; MASH, metabolic dysfunction–associated steatohepatitis; SDI, sociodemographic index.

In 2021, the highest frequencies of MASH-associated PLC incidences (n = 1600), deaths (n = 1300), and DALYs (64,610) in young adults were observed in middle SDI countries (Table 1). The most pronounced ASIR (0.13, 95% UI: 0.10–0.16), ASDR (0.10, 95% UI: 0.08–0.13), and ASDALYs (5.15, 95% UI: 4.00–6.59) per 100,000 population were also observed in middle SDI countries (Figures 4A–C). Between 2000 and 2021, ASIR had the greatest increase in low-middle SDI countries, including ASIR (APC: 1.06%, 95% CI: 1.01%–1.11%), ASDR (APC: 0.98%, 95% CI: 0.91%–1.05%), and ASDALYs (APC: 0.91%, 95% CI: 0.84%–0.98%) (Table 1).

### The burden of MASH-associated PLC in young adults by country

**FIGURE 5:**
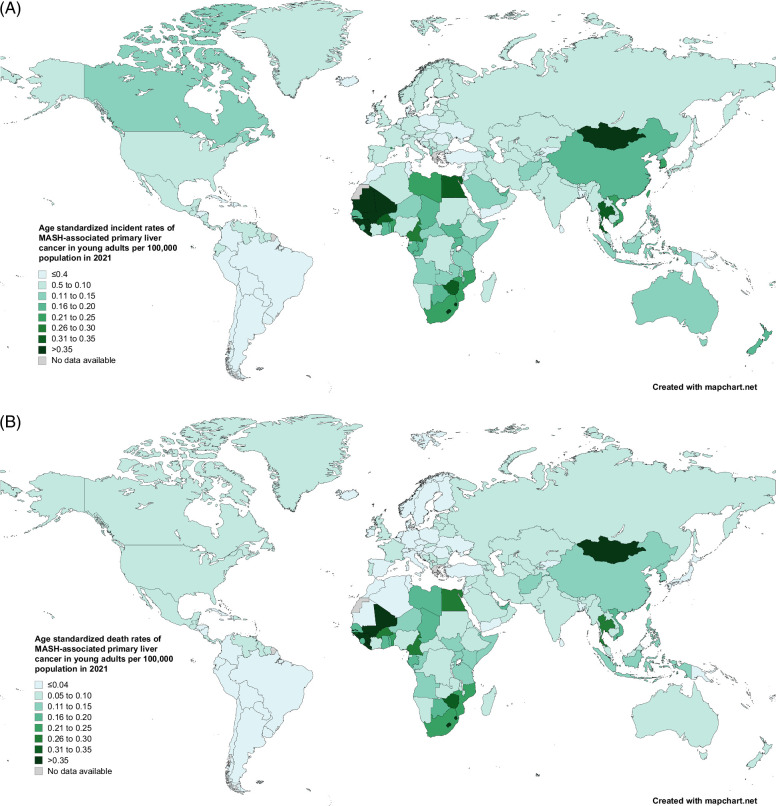
Age-standardized incidence and death rates in 2021 of MASH-associated primary liver cancer in young adults in 2021 by country. (A) Age-standardized incidence rates attributable to MASH-associated primary liver cancer in young adults in 2021 per 100,000 population by country. (B) Age-standardized death rates attributable to MASH-associated primary liver cancer in young adults in 2021 per 100,000 population by country. Abbreviation: MASH, metabolic dysfunction–associated steatohepatitis.

The countries with the highest ASIRs are Mongolia, Gambia, and Eswatini. Mongolia has an ASIR of 0.77 (95% UI: 0.46–1.30), Gambia has an ASIR of 0.66 (95% UI: 0.35–1.16), and Eswatini has an ASIR of 0.62 (95% UI: 0.19–1.55) per 100,000 population (Figure 5A and Supplemental Table S3, http://links.lww.com/HC9/B869). Over half of the 204 countries and territories exhibited an uptrend of ASIR due to MASH-associated PLC, with Poland (APC: 5.74%, 95% CI: 5.52%–5.96%), Uruguay (APC: 4.95%, 95% CI: 4.79%–5.12%), and the United Kingdom (APC: 4.82%,95% CI: 4.66%–4.97%) having the largest increases (Supplemental Table S3, http://links.lww.com/HC9/B869). ASDR of MASH-associated PLC by country in 2021 is shown in Figure 5B and Supplemental Table S4, http://links.lww.com/HC9/B869.

### The burden of MASH-associated PLC in young adults by age group

The ASIR, ASDR, and ASDALYs, along with their APC, for MASH-associated PLC stratified by age among young adults are provided in Supplemental Table S5, http://links.lww.com/HC9/B869. The ASIR of MASH-associated PLC showed an increase in the 15–19 (APC:0.59%, 95% CI: 0.57%–0.62%), 20–24 (APC: 0.41%, 95% CI: 0.30%–0.52%), and 25–29 (APC: 0.18%, 95% CI: 0.06%–0.29%) age groups, while ASDR rose only in the 15–19 (APC: 0.46%, 95% CI: 0.39%–0.53%) and 20–24 (APC: 0.22%, 95% CI: 0.04%–0.40%) age groups. ASDALYs also exhibited an upward trend in the 15–19 (APC: 0.46%, 95% CI: 0.39%–0.53%) and 20–24 (APC: 0.22%, 95% CI: 0.04%–0.40%) age groups, mirroring the ASDR trend.

When compared to younger individuals, the ASIR of individuals over 55 increased more significantly (APC: 0.72%, 95% CI: 0.63%–0.80%). Notably, ASDR and ASDALYs decreased for MASH-associated liver cancer in young adults, while ASDR (APC: 0.57%, 95% CI: 0.43%–0.71%) and ASDALYs (APC: 0.40%, 95% CI: 0.19%–0.61%) increased in those over 55 (Supplemental Table S5, http://links.lww.com/HC9/B869).

## DISCUSSION

Our findings underscore the rising burden of MASH-associated PLC in young adults over the past 2 decades. While the incidence of PLC from most etiologies is declining, MASH is the exception. MASH exhibited the greatest increase in incidence among all etiologies and was the only one with nondecreasing mortality and DALYs from 2000 to 2021. The Eastern Mediterranean region showed the highest increase in MASH-associated PLC incidence and mortality rates in young adults compared to other areas. MASH-associated PLC is increasing at a higher degree in females compared to males. Of notable concern, the incidence rates of MASH-associated PLC in young adults increased in over half of countries and territories.

Previous studies have shown that the overall PLC in young adults is decreasing.^12^^,^^27^ Interestingly, our study revealed that MASH-associated PLC in young adults has increased in the past 2 decades. Continued decline in PLC-related mortality due to hepatitis B or hepatitis C infection due to potent antiviral agents may offset rising rates of PLC-related mortality due to MASLD. A recent study reported that hepatocellular carcinoma-related mortality from MASLD accelerated during the COVID-19 pandemic in the United States.^28^ This increase may be attributed to several factors. First, increased awareness among health care providers may lead to more frequent identification of MASH-associated PLC cases that were previously undiagnosed. Second, the rising prevalence of metabolic disease among young adults is a significant contributor, as these conditions are closely linked to MASH.^16^^,^^17^ Despite this distinct entity receiving less attention than other types of cancer in young adults, no basic science, clinical, or epidemiological studies have been conducted on MASH-associated PLC in this age group.^29^^–^^31^ In addition to preventive measures for metabolic disease and MASLD, further translational and clinical studies should be performed on this unique form of cancer.^30^ Unlike other etiologies of PLC, managing MASLD etiology may be more feasible because a high proportion of MASH-associated PLC cases occur without cirrhosis.^32^ In addition, it is plausible that the incidence of MASH-related PLC will continue to rise in this cohort as they age, given that age is a well-established risk factor.^33^ This emerging risk may potentially offset the reductions in liver cancer associated with the decline in viral hepatitis cases. Consequently, both incidence and mortality rates could increase in the future, suggesting that the current stabilization or decline in rates of overall liver cancer should not be viewed with excessive reassurance.

Despite MASLD and other metabolic diseases being less prevalent in Africa,^16^^,^^17^ the mortality and disability rates were highest in this region. This may be attributed to food insecurity among adolescents and young adults.^34^ Despite the increasing need to address food insecurity, metabolic diseases, MASLD, and MASH, the focus of research in this region continues to be on malnutrition and infectious diseases.^35^^,^^36^ In addition, the persistent burden of PLC from chronic viral hepatitis B and C and ALD, combined with the rapidly rising burden of MASH-associated PLC, will place significant strain on the health care system.^37^ Therefore, the inclusion of food insecurity and genetic differences between MASLD and MASH in the policy should be prioritized to tackle the burden of PLC in this region.

Although women are 3 times less likely to develop PLC and generally have better outcomes than men,^38^ our research indicates that MASH-associated PLC is rising higher in young adult females than males. On the other hand, the prevalence of MASLD has been increased to a higher extent in males (APC 0.80% in males compared to 0.71% in females). The underlying reasons for this remain unexplored. Given that PLC, obesity-associated cancers, and metabolic diseases are generally higher in males, one would expect the burden of MASH-associated PLC, which arises from these risk factors, to also increase in males.^39^^–^^41^ Further studies are needed to examine sex differences, including the influence of sex on noninvasive markers and evidence of sex-specific responses to drugs in clinical trials, particularly in the context of early-onset cancer.

Several limitations inherent to the GBD methodology need to be noted. Variations in data quality across regions may impact the overall findings, especially for rare conditions such as cancer in uncommon age groups. Consequently, the estimation methodology used by the GBD may lead to underestimation, particularly in resource-limited countries.^22^^,^^42^ The GBD approach of attributing PLC to single disease factors may overlook complex causes, such as MASLD combined with increased alcohol consumption, potentially affecting the accuracy of the estimates. Underreporting of alcohol consumption and ALD, which are increasingly prevalent, may result in an underestimation of PLC cases attributed to these factors.^43^^,^^44^ In addition, the GBD was unable to account for confounding factors like obesity, smoking, and comorbidities that may influence the burden of liver cancer.^45^^–^^48^ Lastly, the GBD did not differentiate between histological subtypes like fibrolamellar HCC or cholangiocarcinoma, which may have distinct burdens and trends.^49^

In conclusion, our study highlights that MASH is the only etiology of PLC that has an increasing burden on young adults. As this cohort of patients with MASH ages, the expected continued increases in MASH-related liver cancer may overcome current decreases in viral hepatitis-related PLC. These findings underscore the necessity for a comprehensive strategy addressing the metabolic disease epidemic to prevent cancer in young adults.

## Supplementary Material
