# Supplementary material for: Increased MASH-associated liver cancer in younger demographics
Source: Hepatol Commun. 2025 Jan 7;9(1):e0629. doi: 10.1097/HC9.0000000000000629 (PMC11717512; doi:10.1097/HC9.0000000000000629)
Supplement: Supplementary file 1 [file hc9-9-e0629-s001.docx]

**Supplementary Material 1: Overview of Global Burden of Disease Methodology**

1. Overview

The GBD 2021 estimates mortality and causes of mortality for diseases in 204 countries and territories using a standard methodological approach.

The GBD 2021 uses a variety of data input sources, including surveys, censuses, vital statistics, and other health-related data sources to estimate mortality rates. The input sources are available via the interactive citation tool using the Global Health Data Exchange (GHDx; http://ghdx.healthdata.org/). The user can view and access GHDx records for input sources and export a comma-separated value (CSV) file comprising metadata, citations, and information on where data were used in GBD. Citations for specific GBD components, causes and risks, and locations can be located via this tool. Moreover, the GBD allows the visualisation of its results online. All GBD 2021 online data visualisations are available at https://vizhub.healthdata.org/gbd-compare/, which offers results for all GBD health metrics. The core summary GBD 2021 results comprising mortality data can be obtained in tabular form with the GBD’s data download tool, available at http://ghdx.healthdata.org/gbd‐results-tool. Users must provide an email address, depending on download size, where a download location will be provided to them when the files are ready.

1. Causes of Death Database

Data sources for causes of death were retrieved from vital registration systems, verbal autopsies, and other surveillance systems from 2000-2021. The available data on the cause of death (CoD) are standardised based on the International Classification of Diseases (ICD) 9 and 10 code mapping and pooled into a single database used to generate cause-specific mortality estimates by sex, year, regional geography and age. The CoD database includes seven data sources, including vital registration (VR), verbal autopsy (VA), sibling history, and survey/census. There is no need to use any other data source in countries with complete VR systems. However, less than half of the global population has deaths captured in a VR system. Hence, for these countries with incomplete VR systems, vital statistics for causes of death may be supplemented with other data types^3^. Data inputs used to generate the estimates are found at http://ghdx.healthdata.org/gbd-2021/data-input-sources.

Most of the CoD data is VR data obtained from WHO Mortality Database, a summation of data submitted to WHO by individual countries. VR is obtained from country-specific mortality databases operated by official offices. Whenever possible, each cause is coded directly to the most detailed CoD, whilst cause codes in data tabulated by ICD are coded to aggregated cause groups. Many countries have adopted the ICD Tabulation lists. The ICD tabulation lists include the ICD-9 Basic Tabulation List, the ICD-10 Mortality Tabulation, the Russia Tabulation, and the India Medical Certification of Cause of Death. Sample registration systems are increasingly used in several countries, such as Indonesia and India. In those without VR systems, VA studies are a viable data source to inform CoD. Data are retrieved by trained interviewers who utilise a standardised questionnaire to ask relatives about the signs, symptoms, and demographic characteristics of recently deceased family members. CoD is assigned based on the answers to the questionnaires. Hence, VA data are highly heterogeneous as studies adopt different instruments, cause lists (from single causes to complete ICD-cause lists), methods for assigning CoD, recall periods, and age groups. Cultural differences may also play a part in affecting the interpretation of specific questions. When mapping to the GBD cause, CoD validity might be considered. VAs are likely less accurate for causes requiring medical certification, such as diabetes, than assigning CoD to road injury or homicide.

1. Steps in Data Input

Step 1: Standardise input data

Diverse formats such as mortality databases, literature reviews and reports were used to collect the input data for the cause of death (CoD) database. Usable data sources must demonstrate an apparent sample size of the number of deaths in the population and exhaustive cause lists. The data cleaning process remains complex and differs across data sources; for instance, minimal effort is needed to standardise the data into a consistent structure for VR microdata with location, age, sex, year, and ICD-coded cause of death. The data are subsequently assigned source identifiers to be linked to the GHDx and cited appropriately. Aggregate sex and age categories are then identified for sex-age splitting. Documentation from the source is reviewed to ensure that the population is representative of the location or only a subgroup of the population within that region. Diagnostics were also reviewed at the final stage to avoid sending cleaning errors downstream. All death totals were compared with the sum of cause-specific deaths to ensure that all observed deaths were accounted for and the sample size was complete.

The CoD in tabulated VR data were then compiled into aggregated groups, and some were mapped directly to GBD causes, while others were not informative and thus, cannot be mapped to them. The aggregated causes were mapped and split into ICD detail causes or targets based on the ICD groupings within the aggregated causes. The proportions of deaths from nearby countries with the super-region were used to fill in data gaps as they tended to have similar CoD trends. Global proportions were used for any cause and demographic group for which ICD-detail was lacking.

Step 2: Map to GBD cause list

GBD 2021 used 439 maps to ensure that the cause list from the input data matched the GBD cause list. Examples of the largest and most widely accepted maps used were those of the ICD9 and ICD10 VR data. The mapping process allowed for comparing various data sources across demographic groups.

Step 3: Split Age-sex groups

Some input sources, particularly the VA studies, reported death for a wide range of age groups with different intervals. These different age intervals were then mapped to the GBD standard set of age groups. When the input source contains death due to impossible causes for the given sex and age, it was then redistributed proportionally to all causes.

Step 4: Redistribute

To improve the comparability of the data for CoD, redistribution of uninformative codes, or garbage codes, is needed. Garbage codes to which deaths were assigned should not be considered as the underlying CoD (for example, “heart failure”, “ill-defined cancer site”). For each redistribution package, we defined the “universe” of data as all deaths coded to either the package’s garbage codes or the package’s redistribution targets for each country, year, age, and sex. A regression was run separately for each target group and sex.

Step 5: Correct post-redistribution problems

In this step, data were checked to ensure that the cause list at this point is reasonable given the original data source and how the CoD was assigned. There were two primary corrections that were applied – 1) any cause that is purely an artifact of the redistribution machinery targeting too precise a cause is aggregated up to the parent causes; 2) a “bridge map” is performed over a specific set of sources to ensure that they do not contain causes that could not be reliably determined by the methods used.

Step 6: Drop VR country years or mark them as non‐representative

Lozano and colleagues describe the negative impact of low-completeness VR data on CoD modelling for GBD 2010^4^. For GBD 2021, VR location-years with completeness less than 50% were dropped, and completeness between 50-69% was marked as non-representative. Moreover, any country-year with multiple mortalities registered to major garbage codes greater than 50% of the registered mortality was dropped.

Step 7: Aggregate causes

In this step, the cause list was organised into four levels in a top-down hierarchical format. For example, deaths are classified into three broad groups (Level 1 causes) that include “communicable, maternal, neonatal, nutritional diseases”, “non-communicable diseases”, and “injuries”. Within the Level 1 group of non-communicable diseases is the level 2 cause “diabetes and kidney diseases,” which aggregates the level 3 causes “Diabetes mellitus” and “chronic kidney disease”. “Diabetes mellitus” further aggregates the level 4 causes “Diabetes mellitus type 1” and “Diabetes mellitus type 2”; whilst “chronic kidney disease” aggregates give level 4 causes of “chronic kidney disease due to diabetes type 1”, “chronic kidney disease due to diabetes type 2”, “Hypertensive chronic kidney disease”, “glomerulonephritis chronic kidney disease” and “other chronic kidney disease”. This example demonstrates that the mortality estimate for a parent cause in the hierarchy represents the sum of the deaths due to the causes under that rubric. The parent Level 3 cause estimate includes deaths mapped directly to the parent and any Level 4 sub-causes.

Step 8: Application of noise reduction algorithm

Bayesian noise reduction algorithm was used to deal with zero counts in VR and VA for a specific age group in a specific year. Here, we assume normal prior and a normal data likelihood. Estimating normal prior for the given list of countries was done by running a Poisson regression to estimate the number of deaths due to a specific cause and sex with dummy variables for year and age.

Step 9: Identify outliers in the cause of death

As death rates for CoD tend to have a stable age pattern that does not change rapidly over time in a large population, it is fair to assume a relatively stable pattern in death rate for all causes. Rare exceptions to this include epidemic diseases and specific types of injuries. These outliers have been corrected using the noise reduction process as mentioned in step 8. Identifying outliers occurs before the models’ finalisation for each cause. This is based on the judgment of the modeller and senior faculty, and these outlier decisions are reversible and may be revisited.

1. Causes of Death Modelling Methods

To estimate mortality causes, GBD uses the Cause of Death Ensemble model (CODEm), which combines results from different statistical models weighted based on the out-of-sample predictive validity. The CODEm relies on four key components: First, all available data were identified and gathered to be used in the modelling process. Although these available data may differ in quality, all these data contained some signal of the actual epidemiological process. Second, diverse plausible models are implemented to obtain well-documented associations in the estimates. Using a wide range of individual models to create a predictive ensemble model has outperformed techniques using only a single model both in CoD estimation and in more general prediction applications. Third, the out-of-sample predictive validity is assessed for all individual models, which are then ranked for use in the ensemble modelling stage. Fourth, differently weighted combinations of individual models are examined to select the ensemble model with the highest out-of-sample predictive validity.

A range of plausible statistical models was developed for each cause as several factors may co-vary with any of the CoD. In the CODEm framework, four groups of statistical models are utilized: 1) linear mixed effects regression (LMER) models of the natural log of the cause-specific death rate, 2) LMER models of the logit of the cause fraction, 3) spatiotemporal Gaussian process regression (ST-GPR) models of the natural logarithm of the cause-specific death rate, and 4) SR-GPR models of the logit of the cause fraction. The component models are weighted according to their predictive validity rank to determine their contribution to the ensemble estimate. A set of ensemble models is then determined using the weights.

After the weighting scheme is decided, 1000 draws are created for the final ensemble, with the number of draws contributed by each model proportional to its weight. Mortality estimates were scaled with other causes of death to 100% of all-cause death estimates within each age, sex, year, and location. The estimate for each mortality cause is the mean of 1000 draws from the set best-performing models. 95% uncertainty intervals (UIs) were calculated for all estimates to reflect the 25^th^ and 95^th^ percentile values of the 1000 draws. DALYs were estimated by the summation of years of life lost and years lost due to disability, which serves to quantify the extent of health loss related to specific diseases. Years of life lost were obtained by multiplying the estimated number of deaths by age with a standard life expectancy. In contrast, the multiplication of prevalence computed years lost due to disability by a disability weight, ranging from 0 to 1 where 0 is a state of full health, and 1 is death. Age-standardized rates per 100,000 population were also derived using the direct method to the GBD 2021 population estimate with five-year age groups. All estimates were reported with the corresponding 95% UIs.

**Supplementary Material 2: Sociodemographic index of national and subnational based on the GBD 2021 study.**

(Countries are listed in **bold**, while subnational areas are listed in regular text.)

**Low SDI**

Addis Ababa. **Afghanistan**. Afar. Amhara. Bauchi. **Benin**. Benishangul-Gumuz. Bihar, Rural. Borno. **Burkina Faso**. **Burundi**. Balochistan. **Côte d'Ivoire**. **Central African Republic**. **Chad**. **Democratic Republic of the Congo**. Dire Dawa. **Eritrea**. **Gambia**. Gambella. Gilgit-Baltistan. Gombe. **Guinea**. **Guinea-Bissau**. **Haiti**. Harari. Jharkhand, Rural. Jigawa. Kaduna. Kano. Katsina. Kebbi. Khyber Pakhtunkhwa. **Liberia**. **Madagascar**. **Malawi**. **Mali**. Madhya Pradesh, Rural. **Mozambique**. **Nepal**. **Niger**. Oromia. **Papua New Guinea**. **Rwanda**. **Senegal**. **Sierra Leone**. Sokoto. **Solomon Islands**. **Somalia**. Southern Nations, Nationalities, and Peoples. **South Sudan**. Taraba. **Timor-Leste**. Tigray. **Togo**. **Uganda**. **United Republic of Tanzania**. **Yemen**. Yobe. Zamfara.

**Low Middle SDI**

Acre. Adamawa. Akwa Ibom. Alagoas. Amazonas. Andhra Pradesh, Rural. **Angola**. Arunachal Pradesh, Rural. Assam, Rural. Azad Jammu & Kashmir. Bahia. **Bangladesh**. Baringo. Bayelsa. **Belize**. Bengkulu. Benue. **Bhutan**. Bihar, Urban. **Bolivia (Plurinational State of)**. Bomet. Bungoma. Busia. **Cabo Verde**. **Cambodia**. **Cameroon**. Ceará. Central Java. Central Sulawesi. Chhattisgarh, Rural. **Comoros**. **Congo**. Cross River. Delta. **Democratic People's Republic of Korea**. **Djibouti**. East Nusa Tenggara. Ebonyi. **Egypt**. Ekiti. **El Salvador**. Elgeyo-Marakwet. Embu. Enugu. **Eswatini**. Garissa. **Ghana**. Gorontalo. Guatemala. Gujarat, Rural. Gujarat, Rural. Haryana, Rural. Homa Bay. **Honduras**. Isiolo. Jammu & Kashmir and Ladakh, Rural. Kajiado. Kakamega. Karnataka, Rural. Kericho. Kiambu. Kilifi. **Kiribati**. Kirinyaga. Kisii. Kisumu. Kitui. Kogi. Kwale. Kwara. **Kyrgyzstan**. Laikipia. Lampung. Lamu. **Lao People's Democratic Republic**. **Lesotho**. Machakos. Maharashtra, Rural. Makueni. Maluku. Mandera. Manipur, Rural. Maranhão. Marsabit. **Marshall Islands**. **Mauritania**. Meghalaya, Rural. Meru. **Micronesia (Federated States of)**. Migori. Mizoram, Rural. Mombasa. **Mongolia**. **Morocco**. Murang'a. **Myanmar**. Nagaland, Rural. Nairobi. Nakuru. **Namibia**. Nandi. Narok. Nasarawa. **Nicaragua**. North Maluku. Nyamira. Nyandarua. Nyeri. Odisha, Rural. Ogun. Ondo. Other Union Territories, Rural. Oyo. Paraíba. Pará. Pernambuco. Piauí. Plateau. Punjab. Punjab, Rural. Rajasthan, Rural. Rio Grande do Norte. Rondônia. Roraima. Samburu. **Samoa**. **Sao Tome and Principe**. Sergipe. Siaya. Sikkim, Rural. Sindh. Southeast Sulawesi. **Sudan**. Taita Taveta. **Tajikistan**. Tamil Nadu, Rural. Tana River. Telangana, Rural. Tharaka Nithi. Tibet. Tocantins. Trans Nzoia. Tripura, Rural. Turkana. **Tuvalu**. Uasin Gishu. Uttarakhand, Rural. Uttar Pradesh, Rural. **Vanuatu**. **Venezuela (Bolivarian Republic of**). Vihiga. Wajir. West Bengal, Rural. West Kalimantan. West Nusa Tenggara. West Pokot. West Sulawesi. **Zambia**. **Zimbabwe**.

**Middle SDI**

Abia. Abra. Aceh. Aguascalientes. Agusan Del Norte. Agusan Del Sur. Aklan. **Albania**. Albay. Alborz. **Algeria**. Amapá. Anambra. Andhra Pradesh, Urban. Anhui. Antique. Apayao. Ardebil. **Armenia**. Arunachal Pradesh, Urban. Assam, Urban. **Aurora**. **Azerbaijan**. Baja California. Baja California Sur. Bali. Bangka-Belitung Islands. Banten. Basilan. Bataan. Batanes. Batangas. Benguet. Biliran. Bohol. **Botswana**. Bukidnon. Bulacan. Bushehr. Cagayan. Camarines Norte. Camarines Sur. Camiguin. Campeche. Capiz. Catanduanes. Cavite. Cebu. Central Kalimantan. Chahar Mahaal and Bakhtiari. Chhattisgarh, Urban. Chiapas. Chihuahua. Coahuila. Colima. **Colombia**. **Costa Rica**. Cotabato (North Cotabato). **Cuba**. Davao de Oro. Davao Del Norte. Davao Del Sur. Davao Occidental. Davao Oriental. Delhi, Rural. Dinagat Islands. **Dominican Republic**. Durango. East Azarbayejan. East Java. Eastern Cape. Eastern Samar. **Ecuador**. Edo. **Equatorial Guinea**. Espírito Santo. Fars. FCT (Abuja). **Fiji**. Free State. **Gabon**. Gansu. Gauteng. Gilan. Goa, Rural. Goiás. Golestan. **Grenada**. Guanajuato. Guangxi. Guerrero. Guimaras. Guizhou. Gujarat, Urban. **Guyana**. Hainan. Hamadan. Haryana, Urban. Hebei. Henan. Hidalgo. Himachal Pradesh, Rural. Hormozgan. Hubei. Hunan. Ifugao. Ilam. Ilocos Norte. Ilocos Sur. Iloilo. Imo. **Iraq**. Isabela. Isfahan. Islamabad Capital Territory. Jalisco. **Jamaica**. Jambi. Jammu & Kashmir and Ladakh, Urban. Jharkhand, Urban. Jiangxi. Kalinga. Karnataka, Urban. Kerala. Kerala, Rural. Kerala, Urban. Kerman. Kermanshah. Khorasan-e-Razavi. Khuzestan. Kohgiluyeh and Boyer-Ahmad. Kurdistan. KwaZulu-Natal. La Union. Lagos. Laguna. Lanao Del Norte. Lanao Del Sur. Leyte. Limpopo. Lorestan. Madhya Pradesh, Urban. Maguindanao. Maharashtra, Urban. **Maldives**. Manipur, Urban. Marinduque. Markazi. Masbate. Mato Grosso. Mato Grosso do Sul. Mazandaran. Meghalaya, Urban. Mexico City. Michoacán. Michoacán de Ocampo. Minas Gerais. Misamis Occidental. Misamis Oriental. Mizoram, Urban. Morelos. Mountain Province. Mpumalanga. **México**. Nagaland, Urban. National Capital Region. **Nauru**. Nayarit. Negros Occidental. Negros Oriental. Ningxia. North Khorasan. North Sulawesi. North Sumatra. North-West. Northern Cape. Northern Samar. Nueva Ecija. Nueva Vizcaya. Nuevo León. Oaxaca. Occidental Mindoro. Odisha, Urban. Oriental Mindoro. Osun. Other Union Territories, Urban. Palawan. **Palestine**. Pampanga. **Panama**. Pangasinan. Papua. **Paraguay**. Paraná. **Peru**. Puebla. Punjab, Urban. Qazvin. Qinghai. Qom. Querétaro. Quezon. Quintana Roo. Quirino. Rajasthan, Urban. Rio de Janeiro. Rio Grande do Sul. Rivers. Rizal. Romblon. **Saint Lucia. Saint Vincent and the Grenadines**. Samar (Western Samar). San Luis Potosí. Santa Catarina. Sarangani. Semnan. Shanxi. Sichuan. Sikkim. Sikkim, Urban. Sinaloa. Siquijor. Sistan and Baluchistan. Sonora. Sorsogon. South Cotabato. South Kalimantan. South Khorasan. South Sulawesi. South Sumatra. Southern Leyte. **Sri Lanka**. Sultan Kudarat. Sulu. Surigao Del Norte. Surigao Del Sur. **Suriname**. **Syrian Arab Republic**. São Paulo. Tabasco. Tamaulipas. Tamil Nadu. Tamil Nadu, Urban. Tarlac. Tawi-Tawi. Tehran. Telangana. Telangana, Urban. **Thailand**. Tlaxcala. Tokelau. **Tonga**. Tripura. Tripura, Urban. **Tunisia**. **Turkmenistan**. Uttar Pradesh. Uttar Pradesh, Urban. **Uzbekistan**. Veracruz de Ignacio de la Llave. **Viet Nam**. West Azarbayejan. West Bengal. West Bengal, Urban. West Java. West Papua. West Sumatra. Western Cape. Xinjiang. Yazd. Yogyakarta. Yucatán. Yunnan. Zacatecas. Zambales. Zamboanga Del Norte. Zamboanga Del Sur. Zamboanga Sibugay. Zanjan.

**High Middle SDI**

Abruzzo. Altai Krai. American Samoa. Amur Oblast. **Antigua and Barbuda**. **Argentina**. Arkhangelsk oblast. Arkhangelsk oblast without Nenets autonomous district. Astrakhan Oblast. **Bahamas**. **Bahrain**. **Barbados**. Basilicata. **Belarus**. Belgorod Oblast. **Bosnia and Herzegovina**. **Brunei Darussalam**. Bryansk Oblast. **Bulgaria**. Calabria. Campania. Chechen Republic. Chelyabinsk oblast. **Chile**. Chongqing. Chukotka Autonomous Area. Chukotka Autonomous Okrug. Chuvash Republic. **Cook Islands**. **Croatia**. Delhi, Urban. Distrito Federal. **Dominica**. East Kalimantan. Emilia-Romagna. Friuli-Venezia Giulia. Fujian. **Georgia**. Goa, urban. **Greece**. **Guam**. Guangdong. Heilongjiang. Himachal Pradesh, urban. **Hungary**. Inner Mongolia. Irkutsk Oblast. **Israel**. Ivanovo Oblast. Jakarta. Jewish Autonomous Oblast. Jiangsu. Jilin. **Jordan**. Kabardino-Balkar Republic. Kaliningrad Oblast. Kaluga Oblast. Kamchatka Krai. Karachay-Cherkess Republic. **Kazakhstan**. Kemerovo Oblast. Khabarovsk Krai. Khanty-Mansi autonomous area. Kirov Oblast. Komi Republic. Kostroma Oblast. Krasnodar Krai. Krasnoyarsk Krai. Kurgan Oblast. Kursk Oblast. Lazio. **Lebanon**. Leningrad Oblast. Liaoning. **Libya**. Liguria. Lipetsk Oblast. Lipetzk oblast. Lombardia. Magadan Oblast. **Malaysia**. **Malta**. Marche. **Mauritius**. Molise. **Montenegro**. Moscow City. Moscow Oblast. Murmansk Oblast. Nenets autonomous district. **Niue**. Nizhny Novgorod Oblast. North Kalimantan. **North Macedonia**. Northern Mariana Islands. Novgorod Oblast. Novosibirsk Oblast. **Oman**. Omsk Oblast. Orenburg Oblast. Oryol Oblast. **Palau**. Penza Oblast. Perm Krai. Piemonte. **Portugal**. Primorsky Krai. Provincia autonoma di Bolzano. Provincia autonoma di Trento. Pskov Oblast. Puglia. Republic of Adygeya. Republic of Altai. Republic of Bashkortostan. Republic of Buryatia. Republic of Crimea. Republic of Dagestan. Republic of Ingushetia. Republic of Kalmykia. Republic of Karelia. Republic of Khakassia. Republic of Mari El. **Republic of Moldova**. Republic of Mordovia. Republic of North Ossetia-Alania. Republic of Sakha (Yakutia). Republic of Tatarstan. Republic of Tuva. Riau. Riau Islands. **Romania**. Rostov Oblast. Ryazan Oblast. **Saint Kitts and Nevis**. Saint Petersburg. Sakhalin Oblast. Samara Oblast. Saratov Oblast. Sardegna. **Serbia**. Sevastopol. **Seychelles**. Shaanxi. Shandong. Sicilia. **Slovakia**. Smolensk Oblast. **Spain**. Stavropol Krai. Sverdlovsk Oblast. Tambov Oblast. Tomsk Oblast. Toscana. **Trinidad and Tobago** Tula Oblast. Tver Oblast. Tyumen Oblast. Tyumen oblast without autonomous areas. **Türkiye**. Udmurt Republic. **Ukraine**. **Ukraine (without Crimea & Sevastopol)**. Ulyanovsk Oblast. Umbria. **Uruguay**. Uttarakhand. Uttarakhand, Urban. Valle d'Aosta. Veneto. Vladimir Oblast. Volgograd Oblast. Vologda Oblast. Voronezh Oblast. Yamalo-Nenets autonomous area. Yamalo-Nenets Autonomous Okrug. Yaroslavl Oblast. Zabaikalsk kray. Zhejiang.

**High SDI**

Agder. Aichi. Akita. Alabama. Alaska. **Andorra**. Aomori. Arizona. Arkansas. **Australia**. **Austria**. Barking and Dagenham. Barnet. Barnsley. Bath and North East Somerset. Bedford. Beijing. **Belgium**. Bermuda. Bexley. Birmingham. Blackburn with Darwen. Blackpool. Bolton. Bournemouth. Bracknell Forest. Bradford. Brent. Brighton and Hove. Bristol, City of. Bromley. Buckinghamshire. Bury. Calderdale. California. Cambridgeshire. Camden. **Canada**. Central Bedfordshire. Cheshire East. Cheshire West and Chester. Chiba. Colorado. Connecticut. Cornwall. County Durham. Coventry. Croydon. Cumbria. **Cyprus**. **Czechia**. Darlington. Delaware. **Denmark**. Derby. Derbyshire. Devon. District of Columbia. Dolnośląskie. Doncaster. Dorset. Dudley. Ealing. East Riding of Yorkshire. East Sussex. Ehime. Enfield. Essex. **Estonia**. **Finland**. Florida. **France**. Fukui. Fukuoka. Fukushima. Gateshead. **Georgia.** **Germany**. Gifu. Gloucestershire. **Greenland**. Greenwich. Gunma. Hackney. Halton. Hammersmith and Fulham. Hampshire. Haringey. Harrow. Hartlepool. Havering. Hawaii. Herefordshire, County of. Hertfordshire. Hillingdon. Hiroshima. Hokkaidō. Hong Kong. Hong Kong Special Administrative Region of China. Hounslow. Hyōgo. Ibaraki. **Iceland**. Idaho. Illinois. Indiana. Innlandet. Iowa. **Ireland**. Ishikawa. Isle of Wight. Islington. Iwate. Kagawa. Kagoshima. Kanagawa. Kansas. Kensington and Chelsea. Kent. Kentucky. Kingston upon Hull, City of. Kingston upon Thames. Kirklees. Knowsley. Kujawsko-Pomorskie. Kumamoto. **Kuwait**. Kyoto. Kōchi. Lambeth. Lancashire. **Latvia**. Leeds. Leicester. Leicestershire. Lewisham. Lincolnshire. **Lithuania**. Liverpool. Łódzkie. Louisiana. Lubelskie. Lubuskie. Luton. **Luxembourg**. Macao. Macao Special Administrative Region of China. Maine. Manchester. Maryland. Massachusetts. Mazowieckie. Małopolskie. Medway. Merton. Michigan. Middlesbrough. Mie. Milton Keynes. Minnesota. Mississippi. Missouri. Miyagi. Miyazaki. **Monaco**. Montana. Møre og Romsda. Nagano. Nagasaki. Nara. Nebraska. **Netherlands**. Nevada. New Hampshire. New Jersey. New Mexico. New York. **New Zealand**. New Zealand Maori population. New Zealand non-Maori population. Newcastle upon Tyne. Newham. Niigata. Nordland. Norfolk. North Carolina. North Dakota. North East Lincolnshire. North Lincolnshire. North Somerset. North Tyneside. North Yorkshire. Northamptonshire. **Northern Ireland**. Northumberland. Nottingham. Nottinghamshire. Ohio. Oita. Okayama. Okinawa. Oklahoma. Oldham. Opolskie. Oregon. Osaka. Oslo. Oxfordshire. Pennsylvania. Peterborough. Plymouth. Podkarpackie. Podlaskie. Pomorskie. Poole. Portsmouth. Puerto Rico. **Qatar**. Reading. Redbridge. Redcar and Cleveland. **Republic of Korea**. Rhode Island. Richmond upon Thames. Rochdale. Rogaland. Rotherham. Rutland. Saga. Saitama. Salford. **San Marino**. Sandwell. **Saudi Arabia**. **Scotland**. Sefton. Shanghai. Sheffield. Shiga. Shimane. Shizuoka. Shropshire. **Singapore**. Śląskie. Slough. **Slovenia**. Solihul. Somerset. South Carolina. South Dakota. South Gloucestershire. South Tyneside. Southampton. Southend-on-Sea. Southwark. St Helens. Staffordshire. Stockholm. Stockport. Stockton-on-Tees. Stoke-on-Trent. Suffolk. Sunderland. Surrey. Sutton. **Sweden**. Sweden except Stockholm. Świętokrzyskie. Swindon. **Switzerland**. **Taiwan**. Tameside. Telford and Wrekin. Tennessee. Texas. Thurrock. Tianjin. Tochigi. Tokushima. Tokyo. Torbay. Tottori. Tower Hamlets. Toyama. Trafford. Troms og Finnmark. Trøndelag. **United Arab Emirates**. United States Virgin Islands. Utah. Vermont. Vestfold og Telemark. Vestland. Viken. Virginia. Wakayama. Wakefield. **Wales**. Walsall. Waltham Forest. Wandsworth. Warmińsko-Mazurskie. Warrington. Warwickshire. Washington. West Berkshire. West Sussex. West Virginia. Westminster. Wielkopolskie. Wigan. Wiltshire. Windsor and Maidenhead. Wirral. Wisconsin. Wokingham. Wolverhampton. Worcestershire. Wyoming. Yamagata. Yamaguchi. Yamanashi. York. Zachodniopomorskie.

The subnational sociodemographic Index can be accessed at <https://doi.org/10.1016/S0140-6736(24)00757-8>

**Supplementary Material 3: The burden of MASH-associated primary liver cancer in young adults, compared to other etiologies of liver cancer**

From 2000 to 2021, ASIRs attributable to PLC decreased in HBV (APC: -1.20%, 95% CI -1.39 to -1.02%), HCV (APC: -0.65%, 95% CI -0.73 to -0.57%), and other causes (APC: -1.07%, 95% CI -1.22 to -0.92%), but remained stable from ALD and increased in MASH (APC: 0.26%, 95% CI 0.16 to 0.35%) (**Supplementary Table 1**). ASDR of liver cancer from ALD (APC: -0.38%, 95% CI -0.49 to -0.27%), HBV (APC: -1.78% 95% CI-2.11 to -1.44%), HCV (APC: -0.93%, 95% CI -1.13 to -0.73%), and other causes (APC: -1.50%, 95% CI -1.80 to -1.20%) decreased but remained unchanged in MASH-associated PLC (**Supplementary Table 1**). ASDALYs from ALD (APC: -0.40%, 95% CI -0.54 to -0.27%), HBV (APC: -1.76%, 95% CI -2.16 to -1.36%), HCV (APC: -0.91%, 95% CI -1.08 to -0.75%), and other causes declined (APC: -1.54%, 95% CI -1.86 to -1.23%), but remained stable in MASH-associated PLC (**Supplementary Table 1**).

**Supplementary Table 1 Incidence, Death, Disability-adjusted life years, and age-standardized rates of young adults with five major etiologies of primary liver cancer in 2021 and changes from 2000 to 2021**

|  | **Incidence** |  |  |  | **Death** |  |  |  | **Disability-adjusted life years** |  |  |  |
| --- | --- | --- | --- | --- | --- | --- | --- | --- | --- | --- | --- | --- |
|  | **2021 Number (95% UI)** | **2021 Age-standardized incidence rate (95% UI)** | 2000 to 2021 Annual Percent Change (95% CI) | p | **2021 Number (95% UI)** | **2021 Age-standardized death rate (95% UI)** | 2000 to 2021 Annual Percent Change (95% CI) | p | **2021 Number (95% UI)** | **2021 Age-standardized disability-adjusted life years (95% UI)** | 2000 to 2021 Annual Percent Change (95% CI) | p |
| ALD | 8290 (5770 to 11340) | 0.21 (0.15 to 0.29) | -0.1 (-0.18 to -0.03) | 0.008 | 6590 (4570 to 9040) | 0.17 (0.12 to 0.23) | -0.38 (-0.49 to -0.27) | <0.001 | 312650 (217960 to 424160) | 7.92 (5.52 to 10.74) | -0.4 (-0.54 to -0.27) | <0.001 |
| Hepatitis B | 50880 (42110 to 61740) | 1.29 (1.07 to 1.56) | -1.2 (-1.39 to -1.02) | <0.001 | 39620 (33030 to 47870) | 1 (0.84 to 1.21) | -1.78 (-2.11 to -1.44) | <0.001 | 1956170 (1640490 to 2354430) | 49.54 (41.55 to 59.63) | -1.76 (-2.16 to -1.36) | <0.001 |
| Hepatitis C | 7130 (5430 to 9300) | 0.18 (0.14 to 0.24) | -0.65 (-0.73 to -0.57) | <0.001 | 5650 (4290 to 7440) | 0.14 (0.11 to 0.19) | -0.93 (-1.13 to -0.73) | <0.001 | 265840 (203580 to 349800) | 6.73 (5.16 to 8.86) | -0.91 (-1.08 to -0.75) | <0.001 |
| MASH | 4300 (3310 to 5510) | 0.11 (0.08 to 0.14) | 0.26 (0.16 to 0.35) | <0.001 | 3550 (2730 to 4560) | 0.09 (0.07 to 0.12) | -0.07 (-0.23 to 0.09) | 0.417 | 179340 (140050 to 228420) | 4.54 (3.55 to 5.78) | -0.09 (-0.25 to 0.08) | 0.299 |
| Other causes | 4360 (3380 to 5600) | 0.11 (0.09 to 0.14) | -1.07 (-1.22 to -0.92) | <0.001 | 3420 (2650 to 4410) | 0.09 (0.07 to 0.11) | -1.5 (-1.8 to -1.2) | <0.001 | 175500 (136180 to 226110) | 4.44 (3.45 to 5.73) | -1.54 (-1.86 to -1.23) | <0.001 |

**Abbreviation:** ALD: alcohol-associated liver disease; CI: confidence interval; MASH: metabolic dysfunction-associated steatohepatitis; UI: uncertainty interval

**Supplementary Table 2 Prevalence, Death, Disability-adjusted life years, and age-standardized rates of patients with metabolic dysfunction associated steatotic liver disease in young adults in 2021 and changes from 2000 to 2021**

|  | **Incidence** |  |  |  | **Death** |  |  |  | **Disability-adjusted life years** |  |  |  |
| --- | --- | --- | --- | --- | --- | --- | --- | --- | --- | --- | --- | --- |
|  | 2021 Number (95% UI) | 2021 Age-standardized incidence rate (95% UI) | 2000 to 2021 Annual Percent Change (95% CI) | ***p*** | 2021 Number (95% UI) | 2021 Age-standardized death rate (95% UI) | 2000 to 2021 Annual Percent Change (95% CI) | ***p*** | 2021 Number (95% UI) | 2021 Age-standardized death rate (95% UI) | 2000 to 2021 Annual Percent Change (95% CI) | ***p*** |
| Both | 35604870 (31679960 to 39973200) | 901.71 (802.31 to 1012.34) | 0.76 (0.73 to 0.78) | <0.001 | 18650 (12680 to 26520) | 0.47 (0.32 to 0.67) | 0.44 (0.28 to 0.59) | <0.001 | 929970 (641720 to 1319570) | 23.55 (16.25 to 33.42) | 0.41 (0.27 to 0.56) | <0.001 |
| **By sex** |  |  |  |  |  |  |  |  |  |  |  |  |
| Female | 16097050 (14231000 to 18206170) | 825.97 (730.22 to 934.2) | 0.71 (0.59 to 0.82) | <0.001 | 7880 (5510 to 11020) | 0.4 (0.28 to 0.57) | 0.46 (0.27 to 0.65) | <0.001 | 397540 (282210 to 547990) | 20.4 (14.48 to 28.12) | 0.43 (0.26 to 0.6) | <0.001 |
| Male | 19507820 (17338400 to 21923130) | 975.52 (867.04 to 1096.31) | 0.8 (0.72 to 0.87) | <0.001 | 10770 (7190 to 15700) | 0.54 (0.36 to 0.79) | 0.43 (0.28 to 0.58) | <0.001 | 532430 (360010 to 767210) | 26.63 (18 to 38.37) | 0.41 (0.26 to 0.56) | <0.001 |
| **By WHO region** |  |  |  |  |  |  |  |  |  |  |  |  |
| Africa | 5087520 (4460550 to 5736420) | 909.16 (797.12 to 1025.12) | 0.42 (0.4 to 0.44) | <0.001 | 2360 (1640 to 3360) | 0.42 (0.29 to 0.6) | 0.06 (-0.08 to 0.21) | 0.414 | 122680 (84370 to 173500) | 21.92 (15.08 to 31.01) | 0.04 (-0.1 to 0.17) | 0.6 |
| Eastern Mediterranean | 5769410 (5160180 to 6397990) | 1442.05 (1289.78 to 1599.17) | 0.36 (0.33 to 0.39) | <0.001 | 1440 (1010 to 2040) | 0.36 (0.25 to 0.51) | 0.7 (0.58 to 0.83) | <0.001 | 74090 (52760 to 103410) | 18.52 (13.19 to 25.85) | 0.67 (0.56 to 0.78) | <0.001 |
| Europe | 3173100 (2826040 to 3561300) | 742.24 (661.06 to 833.05) | 0.63 (0.61 to 0.64) | <0.001 | 4100 (2530 to 6240) | 0.96 (0.59 to 1.46) | 1.04 (0.27 to 1.82) | 0.008 | 200700 (124510 to 306050) | 46.95 (29.13 to 71.59) | 1.07 (0.28 to 1.88) | 0.008 |
| Region of the Americas | 4168130 (3706660 to 4677980) | 812.72 (722.74 to 912.13) | 0.64 (0.63 to 0.66) | <0.001 | 4180 (2640 to 6120) | 0.82 (0.51 to 1.19) | 1.17 (0.91 to 1.44) | <0.001 | 204580 (132700 to 297160) | 39.89 (25.88 to 57.94) | 1.14 (0.88 to 1.4) | <0.001 |
| Southeast Asia | 9545470 (8416250 to 10840790) | 848.29 (747.94 to 963.4) | 0.69 (0.66 to 0.72) | <0.001 | 4320 (2880 to 6480) | 0.38 (0.26 to 0.58) | 0.27 (-0.11 to 0.64) | 0.16 | 218850 (146230 to 324950) | 19.45 (13 to 28.88) | 0.15 (-0.26 to 0.56) | 0.464 |
| Western Pacific | 7709290 (6831090 to 8719160) | 849.83 (753.02 to 961.15) | 0.79 (0.71 to 0.87) | <0.001 | 2160 (1600 to 2900) | 0.24 (0.18 to 0.32) | -0.47 (-0.82 to -0.13) | 0.007 | 105210 (78700 to 140280) | 11.6 (8.68 to 15.46) | -0.57 (-0.81 to -0.32) | <0.001 |
| **By Sociodemographic Index** |  |  |  |  |  |  |  |  |  |  |  |  |
| Low SDI | 4608290 (4036990 to 5190210) | 849.64 (744.31 to 956.93) | 0.59 (0.58 to 0.61) | <0.001 | 1740 (1230 to 2410) | 0.32 (0.23 to 0.44) | -0.38 (-0.69 to -0.06) | 0.02 | 90720 (63520 to 125640) | 16.73 (11.71 to 23.16) | -0.4 (-0.71 to -0.09) | 0.012 |
| Low-middle SDI | 9629720 (8516410 to 10852790) | 947.57 (838.02 to 1067.92) | 0.68 (0.67 to 0.69) | <0.001 | 4230 (2900 to 6170) | 0.42 (0.29 to 0.61) | 0.49 (0.42 to 0.57) | <0.001 | 215870 (150440 to 309730) | 21.24 (14.8 to 30.48) | 0.43 (0.35 to 0.5) | <0.001 |
| Middle SDI | 12407110 (11067560 to 13972740) | 988.56 (881.83 to 1113.31) | 0.68 (0.66 to 0.7) | <0.001 | 6650 (4610 to 9380) | 0.53 (0.37 to 0.75) | 1.1 (1 to 1.19) | <0.001 | 329460 (230550 to 459640) | 26.25 (18.37 to 36.62) | 0.99 (0.9 to 1.08) | <0.001 |
| High-middle SDI | 5641490 (5022030 to 6351610) | 896.08 (797.69 to 1008.87) | 0.79 (0.76 to 0.83) | <0.001 | 3780 (2470 to 5680) | 0.6 (0.39 to 0.9) | 1.16 (0.31 to 2.02) | 0.007 | 184620 (123220 to 274300) | 29.32 (19.57 to 43.57) | 1.14 (0.27 to 2.01) | 0.01 |
| High SDI | 3289710 (2932500 to 3694470) | 655.02 (583.89 to 735.61) | 1.04 (1.02 to 1.07) | <0.001 | 2240 (1470 to 3240) | 0.45 (0.29 to 0.65) | -0.79 (-1 to -0.57) | <0.001 | 108420 (71670 to 154650) | 21.59 (14.27 to 30.79) | -0.74 (-1.05 to -0.44) | <0.001 |

**Abbreviation**: CI: confidence interval; SDI: sociodemographic index; UI: uncertainty interval; WHO: World Health Organization

**Supplementary Table 3 Incidence and age-standardized rates of patients with metabolic dysfunction associated steatohepatitis associated primary liver cancer in young adults in 2000 and 2021 and changes from 2000 to 2021**

| **Country** | 2000 Incidence (95% UI) | 2000 Age-standardized incidence rate (95% UI) | 2021 Incidence (95% UI) | 2021 Age-standardized incidence rate (95% UI) | 2000 to 2021 Annual Percent Change (95% CI) | ***p*** |
| --- | --- | --- | --- | --- | --- | --- |
| Afghanistan | 10 (0 to 10) | 0.09 (0.05 to 0.15) | 20 (10 to 30) | 0.11 (0.06 to 0.18) | 1.04 (0.83 to 1.25) | <0.001 |
| Albania | 0 (0 to 0) | 0.11 (0.06 to 0.16) | 0 (0 to 0) | 0.11 (0.06 to 0.18) | 0.16 (-0.11 to 0.43) | 0.252 |
| Algeria | 0 (0 to 10) | 0.02 (0.01 to 0.04) | 10 (10 to 20) | 0.05 (0.03 to 0.08) | 3.23 (3.13 to 3.34) | <0.001 |
| American Samoa | 0 (0 to 0) | 0.16 (0.1 to 0.24) | 0 (0 to 0) | 0.24 (0.13 to 0.38) | 1.96 (1.78 to 2.14) | <0.001 |
| Andorra | 0 (0 to 0) | 0.15 (0.08 to 0.26) | 0 (0 to 0) | 0.21 (0.11 to 0.35) | 1.47 (1.19 to 1.75) | <0.001 |
| Angola | 10 (0 to 30) | 0.13 (0.02 to 0.42) | 20 (0 to 50) | 0.11 (0.03 to 0.31) | -0.55 (-0.64 to -0.46) | <0.001 |
| Antigua and Barbuda | 0 (0 to 0) | 0.03 (0.02 to 0.05) | 0 (0 to 0) | 0.03 (0.02 to 0.05) | 0.38 (0.04 to 0.72) | 0.029 |
| Argentina | 0 (0 to 0) | 0.01 (0.01 to 0.01) | 0 (0 to 10) | 0.02 (0.01 to 0.03) | 3.34 (3.25 to 3.42) | <0.001 |
| Armenia | 0 (0 to 0) | 0.08 (0.05 to 0.12) | 0 (0 to 0) | 0.09 (0.06 to 0.13) | 0.47 (0.11 to 0.84) | 0.011 |
| Australia | 10 (0 to 10) | 0.06 (0.04 to 0.09) | 20 (10 to 20) | 0.13 (0.09 to 0.19) | 3.82 (3.7 to 3.94) | <0.001 |
| Austria | 0 (0 to 0) | 0.04 (0.03 to 0.06) | 0 (0 to 0) | 0.06 (0.04 to 0.09) | 1.75 (1.65 to 1.85) | <0.001 |
| Azerbaijan | 0 (0 to 10) | 0.09 (0.05 to 0.15) | 10 (0 to 10) | 0.11 (0.05 to 0.21) | 0.96 (0.8 to 1.11) | <0.001 |
| Bahamas | 0 (0 to 0) | 0.06 (0.04 to 0.09) | 0 (0 to 0) | 0.07 (0.05 to 0.11) | 0.68 (0.43 to 0.93) | <0.001 |
| Bahrain | 0 (0 to 0) | 0.07 (0.04 to 0.11) | 0 (0 to 0) | 0.07 (0.04 to 0.11) | -0.29 (-0.73 to 0.15) | 0.191 |
| Bangladesh | 20 (10 to 30) | 0.03 (0.02 to 0.05) | 30 (20 to 60) | 0.04 (0.02 to 0.06) | 1.74 (1.63 to 1.84) | <0.001 |
| Barbados | 0 (0 to 0) | 0.05 (0.03 to 0.07) | 0 (0 to 0) | 0.05 (0.03 to 0.08) | 0.29 (0.21 to 0.36) | <0.001 |
| Belarus | 0 (0 to 0) | 0.04 (0.02 to 0.06) | 0 (0 to 0) | 0.05 (0.03 to 0.09) | 1.82 (1.54 to 2.11) | <0.001 |
| Belgium | 0 (0 to 0) | 0.04 (0.02 to 0.05) | 0 (0 to 0) | 0.05 (0.03 to 0.07) | 1.19 (1.11 to 1.27) | <0.001 |
| Belize | 0 (0 to 0) | 0.04 (0.03 to 0.05) | 0 (0 to 0) | 0.05 (0.03 to 0.07) | 1.27 (1.02 to 1.53) | <0.001 |
| Benin | 10 (0 to 20) | 0.36 (0.17 to 0.64) | 20 (10 to 30) | 0.25 (0.14 to 0.43) | -1.64 (-1.9 to -1.37) | <0.001 |
| Bermuda | 0 (0 to 0) | 0.03 (0.02 to 0.05) | 0 (0 to 0) | 0.04 (0.02 to 0.06) | 0.87 (0.62 to 1.12) | <0.001 |
| Bhutan | 0 (0 to 0) | 0.06 (0.03 to 0.1) | 0 (0 to 0) | 0.08 (0.04 to 0.14) | 1.52 (1.4 to 1.64) | <0.001 |
| Bolivia (Plurinational State of) | 0 (0 to 0) | 0.04 (0.02 to 0.06) | 0 (0 to 0) | 0.04 (0.02 to 0.07) | 0.66 (0.56 to 0.76) | <0.001 |
| Bosnia and Herzegovina | 0 (0 to 0) | 0.09 (0.05 to 0.14) | 0 (0 to 0) | 0.06 (0.04 to 0.11) | -1.44 (-1.66 to -1.22) | <0.001 |
| Botswana | 0 (0 to 0) | 0.16 (0.04 to 0.48) | 0 (0 to 10) | 0.17 (0.07 to 0.42) | 0.47 (0.29 to 0.64) | <0.001 |
| Brazil | 20 (20 to 30) | 0.02 (0.02 to 0.03) | 40 (30 to 40) | 0.03 (0.03 to 0.04) | 1.41 (1.27 to 1.55) | <0.001 |
| Brunei Darussalam | 0 (0 to 0) | 0.06 (0.03 to 0.1) | 0 (0 to 0) | 0.07 (0.04 to 0.13) | 0.94 (0.62 to 1.26) | <0.001 |
| Bulgaria | 0 (0 to 0) | 0.08 (0.05 to 0.12) | 0 (0 to 0) | 0.07 (0.04 to 0.11) | -0.49 (-0.88 to -0.1) | 0.014 |
| Burkina Faso | 20 (10 to 40) | 0.33 (0.13 to 0.75) | 30 (10 to 70) | 0.33 (0.13 to 0.69) | -0.1 (-0.26 to 0.05) | 0.18 |
| Burundi | 0 (0 to 0) | 0.1 (0.05 to 0.17) | 0 (0 to 10) | 0.06 (0.03 to 0.12) | -1.8 (-1.87 to -1.73) | <0.001 |
| Cote d'Ivoire | 10 (0 to 10) | 0.09 (0.05 to 0.14) | 10 (10 to 20) | 0.07 (0.04 to 0.13) | -0.83 (-1.03 to -0.64) | <0.001 |
| Cabo Verde | 0 (0 to 0) | 0.25 (0.14 to 0.41) | 0 (0 to 0) | 0.31 (0.16 to 0.51) | 1.01 (0.86 to 1.16) | <0.001 |
| Cambodia | 10 (0 to 10) | 0.11 (0.05 to 0.25) | 10 (0 to 20) | 0.1 (0.05 to 0.21) | -0.52 (-0.56 to -0.48) | <0.001 |
| Cameroon | 20 (10 to 40) | 0.36 (0.2 to 0.63) | 50 (20 to 80) | 0.3 (0.15 to 0.53) | -0.97 (-1.09 to -0.84) | <0.001 |
| Canada | 10 (10 to 20) | 0.08 (0.05 to 0.12) | 20 (10 to 30) | 0.12 (0.08 to 0.17) | 1.91 (1.76 to 2.06) | <0.001 |
| Central African Republic | 0 (0 to 10) | 0.15 (0.06 to 0.35) | 0 (0 to 10) | 0.11 (0.04 to 0.23) | -1.58 (-1.73 to -1.43) | <0.001 |
| Chad | 10 (0 to 20) | 0.26 (0.11 to 0.53) | 10 (10 to 30) | 0.19 (0.09 to 0.38) | -1.41 (-1.56 to -1.26) | <0.001 |
| Chile | 0 (0 to 0) | 0.02 (0.01 to 0.02) | 0 (0 to 0) | 0.03 (0.02 to 0.05) | 3.28 (3.24 to 3.32) | <0.001 |
| China | 1320 (1030 to 1660) | 0.18 (0.14 to 0.23) | 1260 (930 to 1670) | 0.19 (0.14 to 0.25) | 0.17 (-0.15 to 0.5) | 0.299 |
| Colombia | 10 (0 to 10) | 0.03 (0.02 to 0.04) | 10 (10 to 10) | 0.04 (0.02 to 0.05) | 1.64 (1.42 to 1.85) | <0.001 |
| Comoros | 0 (0 to 0) | 0.13 (0.07 to 0.21) | 0 (0 to 0) | 0.14 (0.08 to 0.24) | 0.52 (0.35 to 0.7) | <0.001 |
| Congo | 0 (0 to 10) | 0.17 (0.07 to 0.4) | 0 (0 to 10) | 0.16 (0.06 to 0.37) | -0.43 (-0.54 to -0.32) | <0.001 |
| Cook Islands | 0 (0 to 0) | 0.25 (0.15 to 0.38) | 0 (0 to 0) | 0.35 (0.19 to 0.56) | 1.67 (1.43 to 1.92) | <0.001 |
| Costa Rica | 0 (0 to 0) | 0.07 (0.05 to 0.1) | 0 (0 to 0) | 0.1 (0.07 to 0.15) | 1.82 (1.68 to 1.95) | <0.001 |
| Croatia | 0 (0 to 0) | 0.04 (0.02 to 0.07) | 0 (0 to 0) | 0.03 (0.02 to 0.05) | -1.45 (-1.94 to -0.95) | <0.001 |
| Cuba | 0 (0 to 0) | 0.04 (0.02 to 0.05) | 0 (0 to 0) | 0.04 (0.03 to 0.06) | 0.51 (0.17 to 0.86) | 0.003 |
| Cyprus | 0 (0 to 0) | 0.03 (0.02 to 0.04) | 0 (0 to 0) | 0.03 (0.01 to 0.04) | -0.41 (-0.5 to -0.31) | <0.001 |
| Czechia | 0 (0 to 0) | 0.04 (0.02 to 0.06) | 0 (0 to 0) | 0.03 (0.02 to 0.04) | -1.68 (-1.85 to -1.5) | <0.001 |
| Democratic People's Republic of Korea | 20 (10 to 40) | 0.16 (0.07 to 0.3) | 20 (10 to 40) | 0.15 (0.08 to 0.27) | -0.5 (-0.59 to -0.4) | <0.001 |
| Democratic Republic of the Congo | 10 (10 to 30) | 0.06 (0.02 to 0.13) | 20 (10 to 50) | 0.05 (0.02 to 0.12) | -0.38 (-0.51 to -0.25) | <0.001 |
| Denmark | 0 (0 to 0) | 0.03 (0.02 to 0.04) | 0 (0 to 0) | 0.02 (0.01 to 0.03) | -1.88 (-2.23 to -1.54) | <0.001 |
| Djibouti | 0 (0 to 0) | 0.09 (0.05 to 0.16) | 0 (0 to 0) | 0.11 (0.06 to 0.19) | 1.05 (0.96 to 1.14) | <0.001 |
| Dominica | 0 (0 to 0) | 0.04 (0.03 to 0.07) | 0 (0 to 0) | 0.05 (0.03 to 0.09) | 1.03 (0.89 to 1.16) | <0.001 |
| Dominican Republic | 0 (0 to 0) | 0.02 (0.01 to 0.03) | 0 (0 to 0) | 0.04 (0.02 to 0.06) | 3.02 (2.77 to 3.27) | <0.001 |
| Ecuador | 0 (0 to 0) | 0.03 (0.02 to 0.05) | 0 (0 to 10) | 0.05 (0.03 to 0.07) | 2.09 (1.53 to 2.65) | <0.001 |
| Egypt | 80 (50 to 120) | 0.23 (0.13 to 0.35) | 180 (110 to 260) | 0.33 (0.2 to 0.48) | 1.86 (1.77 to 1.96) | <0.001 |
| El Salvador | 0 (0 to 0) | 0.02 (0.02 to 0.03) | 0 (0 to 0) | 0.04 (0.02 to 0.06) | 2.41 (2.04 to 2.77) | <0.001 |
| Equatorial Guinea | 0 (0 to 0) | 0.05 (0.02 to 0.1) | 0 (0 to 0) | 0.1 (0.05 to 0.17) | 3.13 (2.88 to 3.38) | <0.001 |
| Eritrea | 0 (0 to 0) | 0.09 (0.05 to 0.17) | 0 (0 to 10) | 0.09 (0.05 to 0.16) | -0.15 (-0.22 to -0.08) | <0.001 |
| Estonia | 0 (0 to 0) | 0.06 (0.04 to 0.09) | 0 (0 to 0) | 0.06 (0.04 to 0.08) | -0.26 (-0.42 to -0.11) | 0.001 |
| Eswatini | 0 (0 to 0) | 0.57 (0.29 to 0.99) | 0 (0 to 10) | 0.62 (0.19 to 1.55) | 0.4 (0.18 to 0.63) | <0.001 |
| Ethiopia | 20 (10 to 30) | 0.06 (0.05 to 0.09) | 30 (20 to 40) | 0.05 (0.03 to 0.08) | -1.15 (-1.32 to -0.98) | <0.001 |
| Fiji | 0 (0 to 0) | 0.07 (0.04 to 0.11) | 0 (0 to 0) | 0.12 (0.06 to 0.2) | 2.52 (2.19 to 2.84) | <0.001 |
| Finland | 0 (0 to 0) | 0.05 (0.03 to 0.08) | 0 (0 to 0) | 0.05 (0.03 to 0.08) | 0.1 (-0.23 to 0.43) | 0.538 |
| France | 20 (10 to 30) | 0.06 (0.04 to 0.09) | 20 (10 to 30) | 0.08 (0.05 to 0.12) | 1.13 (0.84 to 1.43) | <0.001 |
| Gabon | 0 (0 to 0) | 0.19 (0.08 to 0.4) | 0 (0 to 0) | 0.2 (0.09 to 0.36) | 0.14 (-0.01 to 0.28) | 0.059 |
| Gambia | 0 (0 to 10) | 0.54 (0.32 to 0.83) | 10 (0 to 10) | 0.66 (0.35 to 1.16) | 0.92 (0.84 to 1) | <0.001 |
| Georgia | 0 (0 to 0) | 0.06 (0.04 to 0.08) | 0 (0 to 0) | 0.06 (0.04 to 0.09) | 0.39 (0 to 0.78) | 0.051 |
| Germany | 20 (10 to 30) | 0.04 (0.03 to 0.06) | 20 (10 to 30) | 0.05 (0.03 to 0.08) | 0.93 (0.73 to 1.13) | <0.001 |
| Ghana | 30 (20 to 50) | 0.3 (0.18 to 0.52) | 30 (20 to 60) | 0.2 (0.1 to 0.34) | -2.05 (-2.19 to -1.9) | <0.001 |
| Greece | 0 (0 to 0) | 0.03 (0.02 to 0.05) | 0 (0 to 0) | 0.06 (0.04 to 0.08) | 2.89 (2.77 to 3) | <0.001 |
| Greenland | 0 (0 to 0) | 0.11 (0.06 to 0.18) | 0 (0 to 0) | 0.09 (0.05 to 0.15) | -0.89 (-1.04 to -0.73) | <0.001 |
| Grenada | 0 (0 to 0) | 0.03 (0.02 to 0.04) | 0 (0 to 0) | 0.06 (0.03 to 0.08) | 3.07 (2.78 to 3.37) | <0.001 |
| Guam | 0 (0 to 0) | 0.09 (0.06 to 0.14) | 0 (0 to 0) | 0.18 (0.11 to 0.28) | 3.13 (3.06 to 3.2) | <0.001 |
| Guatemala | 0 (0 to 0) | 0.05 (0.04 to 0.07) | 10 (0 to 10) | 0.09 (0.06 to 0.12) | 2.49 (2.2 to 2.77) | <0.001 |
| Guinea | 20 (10 to 20) | 0.45 (0.27 to 0.73) | 30 (10 to 40) | 0.42 (0.22 to 0.69) | -0.37 (-0.45 to -0.29) | <0.001 |
| Guinea-Bissau | 0 (0 to 0) | 0.49 (0.21 to 0.88) | 0 (0 to 10) | 0.37 (0.2 to 0.66) | -1.26 (-1.37 to -1.15) | <0.001 |
| Guyana | 0 (0 to 0) | 0.05 (0.03 to 0.07) | 0 (0 to 0) | 0.05 (0.03 to 0.08) | 0.37 (0.11 to 0.63) | 0.005 |
| Haiti | 0 (0 to 0) | 0.02 (0.01 to 0.04) | 0 (0 to 0) | 0.02 (0.01 to 0.04) | -0.26 (-0.38 to -0.15) | <0.001 |
| Honduras | 0 (0 to 0) | 0.04 (0.02 to 0.06) | 0 (0 to 0) | 0.04 (0.02 to 0.07) | 0.52 (0.46 to 0.59) | <0.001 |
| Hungary | 0 (0 to 0) | 0.04 (0.03 to 0.07) | 0 (0 to 0) | 0.02 (0.01 to 0.04) | -2.72 (-3.29 to -2.15) | <0.001 |
| Iceland | 0 (0 to 0) | 0.03 (0.02 to 0.05) | 0 (0 to 0) | 0.04 (0.03 to 0.06) | 1.3 (1.14 to 1.45) | <0.001 |
| India | 280 (230 to 340) | 0.05 (0.04 to 0.06) | 530 (410 to 670) | 0.07 (0.05 to 0.09) | 1.16 (1.03 to 1.29) | <0.001 |
| Indonesia | 100 (60 to 140) | 0.08 (0.05 to 0.12) | 180 (110 to 280) | 0.12 (0.07 to 0.19) | 1.6 (1.54 to 1.66) | <0.001 |
| Iran (Islamic Republic of) | 10 (10 to 20) | 0.04 (0.03 to 0.05) | 30 (20 to 40) | 0.06 (0.05 to 0.08) | 2.58 (2.12 to 3.05) | <0.001 |
| Iraq | 10 (0 to 10) | 0.05 (0.03 to 0.09) | 20 (10 to 30) | 0.08 (0.05 to 0.14) | 2.07 (1.89 to 2.24) | <0.001 |
| Ireland | 0 (0 to 0) | 0.03 (0.02 to 0.04) | 0 (0 to 0) | 0.04 (0.02 to 0.06) | 1.96 (1.86 to 2.06) | <0.001 |
| Israel | 0 (0 to 0) | 0.03 (0.02 to 0.04) | 0 (0 to 0) | 0.03 (0.02 to 0.04) | 0.36 (0.27 to 0.45) | <0.001 |
| Italy | 10 (10 to 20) | 0.05 (0.04 to 0.06) | 10 (10 to 20) | 0.05 (0.04 to 0.06) | -0.16 (-0.65 to 0.33) | 0.514 |
| Jamaica | 0 (0 to 0) | 0.02 (0.01 to 0.03) | 0 (0 to 0) | 0.03 (0.02 to 0.05) | 1.5 (1.18 to 1.82) | <0.001 |
| Japan | 60 (50 to 80) | 0.1 (0.08 to 0.13) | 30 (20 to 30) | 0.05 (0.04 to 0.06) | -3.24 (-3.34 to -3.14) | <0.001 |
| Jordan | 0 (0 to 0) | 0.02 (0.01 to 0.04) | 0 (0 to 0) | 0.03 (0.02 to 0.04) | 0.74 (0.58 to 0.9) | <0.001 |
| Kazakhstan | 10 (10 to 20) | 0.17 (0.11 to 0.25) | 10 (10 to 10) | 0.08 (0.05 to 0.12) | -3.52 (-4.18 to -2.85) | <0.001 |
| Kenya | 10 (10 to 20) | 0.08 (0.05 to 0.13) | 30 (20 to 50) | 0.13 (0.09 to 0.19) | 2.24 (2.12 to 2.36) | <0.001 |
| Kiribati | 0 (0 to 0) | 0.2 (0.12 to 0.3) | 0 (0 to 0) | 0.17 (0.1 to 0.29) | -0.61 (-0.78 to -0.44) | <0.001 |
| Kuwait | 0 (0 to 0) | 0.04 (0.02 to 0.05) | 0 (0 to 0) | 0.02 (0.01 to 0.03) | -2.17 (-2.29 to -2.04) | <0.001 |
| Kyrgyzstan | 0 (0 to 0) | 0.04 (0.02 to 0.06) | 0 (0 to 0) | 0.03 (0.02 to 0.05) | -1.11 (-1.34 to -0.89) | <0.001 |
| Lao People's Democratic Republic | 0 (0 to 10) | 0.12 (0.07 to 0.21) | 0 (0 to 10) | 0.1 (0.05 to 0.18) | -0.83 (-0.89 to -0.77) | <0.001 |
| Latvia | 0 (0 to 0) | 0.05 (0.03 to 0.07) | 0 (0 to 0) | 0.05 (0.03 to 0.08) | 0.59 (0.4 to 0.79) | <0.001 |
| Lebanon | 0 (0 to 0) | 0.03 (0.02 to 0.05) | 0 (0 to 0) | 0.03 (0.02 to 0.06) | 0.8 (0.41 to 1.18) | <0.001 |
| Lesotho | 0 (0 to 10) | 0.27 (0.12 to 0.62) | 0 (0 to 10) | 0.43 (0.15 to 1.16) | 2.2 (2.09 to 2.3) | <0.001 |
| Liberia | 0 (0 to 10) | 0.31 (0.14 to 0.61) | 10 (10 to 20) | 0.4 (0.21 to 0.69) | 1.21 (1.03 to 1.4) | <0.001 |
| Libya | 0 (0 to 0) | 0.09 (0.05 to 0.14) | 10 (0 to 10) | 0.21 (0.12 to 0.35) | 4.13 (3.94 to 4.33) | <0.001 |
| Lithuania | 0 (0 to 0) | 0.04 (0.02 to 0.06) | 0 (0 to 0) | 0.06 (0.04 to 0.09) | 1.91 (1.58 to 2.24) | <0.001 |
| Luxembourg | 0 (0 to 0) | 0.04 (0.02 to 0.06) | 0 (0 to 0) | 0.04 (0.02 to 0.06) | 0.07 (-0.06 to 0.19) | 0.282 |
| Madagascar | 10 (0 to 10) | 0.07 (0.04 to 0.12) | 10 (10 to 20) | 0.08 (0.04 to 0.12) | 0.16 (0.13 to 0.18) | <0.001 |
| Malawi | 10 (0 to 10) | 0.12 (0.07 to 0.21) | 10 (10 to 20) | 0.11 (0.06 to 0.18) | -0.49 (-0.58 to -0.41) | <0.001 |
| Malaysia | 10 (10 to 10) | 0.07 (0.05 to 0.11) | 20 (10 to 30) | 0.1 (0.06 to 0.16) | 1.24 (1.18 to 1.3) | <0.001 |
| Maldives | 0 (0 to 0) | 0.04 (0.02 to 0.06) | 0 (0 to 0) | 0.04 (0.02 to 0.08) | 0.61 (0.21 to 1.01) | 0.003 |
| Mali | 20 (10 to 30) | 0.36 (0.22 to 0.58) | 40 (20 to 70) | 0.39 (0.22 to 0.64) | 0.34 (0.18 to 0.5) | <0.001 |
| Malta | 0 (0 to 0) | 0.02 (0.02 to 0.04) | 0 (0 to 0) | 0.03 (0.02 to 0.05) | 1.45 (1.37 to 1.52) | <0.001 |
| Marshall Islands | 0 (0 to 0) | 0.08 (0.04 to 0.13) | 0 (0 to 0) | 0.1 (0.05 to 0.18) | 1.27 (1.19 to 1.36) | <0.001 |
| Mauritania | 10 (0 to 10) | 0.53 (0.16 to 1.2) | 10 (0 to 10) | 0.39 (0.18 to 0.7) | -1.59 (-1.75 to -1.42) | <0.001 |
| Mauritius | 0 (0 to 0) | 0.02 (0.02 to 0.04) | 0 (0 to 0) | 0.02 (0.01 to 0.02) | -2.16 (-2.44 to -1.89) | <0.001 |
| Mexico | 20 (20 to 20) | 0.03 (0.03 to 0.04) | 40 (30 to 40) | 0.05 (0.04 to 0.06) | 2.09 (2.01 to 2.17) | <0.001 |
| Micronesia (Federated States of) | 0 (0 to 0) | 0.14 (0.07 to 0.25) | 0 (0 to 0) | 0.14 (0.07 to 0.24) | 0.01 (-0.1 to 0.12) | 0.908 |
| Monaco | 0 (0 to 0) | 0.11 (0.07 to 0.19) | 0 (0 to 0) | 0.16 (0.09 to 0.27) | 1.6 (1.38 to 1.82) | <0.001 |
| Mongolia | 10 (0 to 10) | 0.64 (0.35 to 1.09) | 10 (10 to 20) | 0.77 (0.46 to 1.3) | 0.91 (0.79 to 1.03) | <0.001 |
| Montenegro | 0 (0 to 0) | 0.1 (0.06 to 0.15) | 0 (0 to 0) | 0.1 (0.06 to 0.16) | 0.12 (-0.12 to 0.36) | 0.326 |
| Morocco | 0 (0 to 0) | 0.01 (0 to 0.01) | 0 (0 to 0) | 0.01 (0.01 to 0.02) | 2.73 (2.58 to 2.88) | <0.001 |
| Mozambique | 20 (10 to 20) | 0.19 (0.11 to 0.31) | 30 (10 to 70) | 0.23 (0.1 to 0.46) | 0.84 (0.75 to 0.92) | <0.001 |
| Myanmar | 10 (0 to 30) | 0.06 (0.02 to 0.12) | 20 (10 to 40) | 0.06 (0.02 to 0.13) | 0.24 (0.17 to 0.31) | <0.001 |
| Namibia | 0 (0 to 0) | 0.06 (0.04 to 0.1) | 0 (0 to 0) | 0.06 (0.03 to 0.11) | -0.1 (-0.27 to 0.07) | 0.246 |
| Nauru | 0 (0 to 0) | 0.18 (0.1 to 0.32) | 0 (0 to 0) | 0.18 (0.09 to 0.31) | -0.05 (-0.14 to 0.03) | 0.206 |
| Nepal | 0 (0 to 10) | 0.03 (0.02 to 0.05) | 10 (10 to 20) | 0.06 (0.03 to 0.1) | 3.4 (3.1 to 3.69) | <0.001 |
| Netherlands | 0 (0 to 0) | 0.02 (0.02 to 0.03) | 0 (0 to 0) | 0.03 (0.02 to 0.04) | 1.06 (0.97 to 1.15) | <0.001 |
| New Zealand | 0 (0 to 0) | 0.11 (0.09 to 0.13) | 0 (0 to 10) | 0.18 (0.15 to 0.23) | 2.4 (2.21 to 2.6) | <0.001 |
| Nicaragua | 0 (0 to 0) | 0.03 (0.02 to 0.04) | 0 (0 to 0) | 0.05 (0.03 to 0.08) | 2.23 (2.1 to 2.36) | <0.001 |
| Niger | 10 (10 to 20) | 0.24 (0.1 to 0.51) | 10 (10 to 30) | 0.14 (0.07 to 0.26) | -2.41 (-2.5 to -2.31) | <0.001 |
| Nigeria | 50 (20 to 100) | 0.09 (0.04 to 0.17) | 80 (40 to 140) | 0.07 (0.04 to 0.13) | -0.8 (-0.97 to -0.63) | <0.001 |
| Niue | 0 (0 to 0) | 0.14 (0.07 to 0.23) | 0 (0 to 0) | 0.14 (0.08 to 0.26) | 0.25 (0.16 to 0.34) | <0.001 |
| North Macedonia | 0 (0 to 0) | 0.1 (0.06 to 0.16) | 0 (0 to 0) | 0.09 (0.05 to 0.15) | -0.53 (-0.76 to -0.31) | <0.001 |
| Northern Mariana Islands | 0 (0 to 0) | 0.13 (0.07 to 0.21) | 0 (0 to 0) | 0.15 (0.08 to 0.26) | 0.4 (-0.58 to 1.38) | 0.428 |
| Norway | 0 (0 to 0) | 0.03 (0.03 to 0.04) | 0 (0 to 0) | 0.07 (0.05 to 0.08) | 3.36 (3.09 to 3.63) | <0.001 |
| Oman | 0 (0 to 0) | 0.04 (0.02 to 0.07) | 0 (0 to 0) | 0.06 (0.04 to 0.11) | 2.15 (2.05 to 2.25) | <0.001 |
| Pakistan | 50 (40 to 60) | 0.07 (0.06 to 0.09) | 100 (80 to 140) | 0.09 (0.06 to 0.11) | 0.74 (0.67 to 0.81) | <0.001 |
| Palau | 0 (0 to 0) | 0.23 (0.11 to 0.41) | 0 (0 to 0) | 0.31 (0.16 to 0.57) | 1.49 (1.11 to 1.87) | <0.001 |
| Palestine | 0 (0 to 0) | 0.06 (0.03 to 0.09) | 0 (0 to 0) | 0.06 (0.04 to 0.09) | 0.21 (0.05 to 0.37) | 0.009 |
| Panama | 0 (0 to 0) | 0.03 (0.02 to 0.04) | 0 (0 to 0) | 0.06 (0.03 to 0.08) | 3.31 (3.15 to 3.48) | <0.001 |
| Papua New Guinea | 0 (0 to 0) | 0.05 (0.02 to 0.12) | 0 (0 to 10) | 0.04 (0.02 to 0.1) | -1.05 (-1.1 to -0.99) | <0.001 |
| Paraguay | 0 (0 to 0) | 0.02 (0.01 to 0.04) | 0 (0 to 0) | 0.04 (0.02 to 0.06) | 2.59 (2.33 to 2.85) | <0.001 |
| Peru | 0 (0 to 10) | 0.03 (0.02 to 0.04) | 10 (0 to 10) | 0.04 (0.02 to 0.06) | 1.8 (1.59 to 2.01) | <0.001 |
| Philippines | 50 (40 to 70) | 0.13 (0.09 to 0.16) | 70 (50 to 90) | 0.12 (0.09 to 0.15) | -0.43 (-0.49 to -0.36) | <0.001 |
| Poland | 0 (0 to 0) | 0.01 (0.01 to 0.01) | 10 (0 to 10) | 0.03 (0.02 to 0.03) | 5.74 (5.52 to 5.96) | <0.001 |
| Portugal | 0 (0 to 0) | 0.04 (0.03 to 0.06) | 0 (0 to 0) | 0.06 (0.04 to 0.09) | 1.86 (1.62 to 2.11) | <0.001 |
| Puerto Rico | 0 (0 to 0) | 0.05 (0.03 to 0.08) | 0 (0 to 0) | 0.09 (0.05 to 0.13) | 2.37 (2.25 to 2.49) | <0.001 |
| Qatar | 0 (0 to 0) | 0.15 (0.09 to 0.25) | 0 (0 to 10) | 0.15 (0.08 to 0.23) | -0.38 (-0.63 to -0.12) | 0.004 |
| Republic of Korea | 70 (40 to 110) | 0.26 (0.16 to 0.4) | 50 (30 to 90) | 0.21 (0.12 to 0.35) | -0.92 (-1.11 to -0.73) | <0.001 |
| Republic of Moldova | 0 (0 to 0) | 0.05 (0.03 to 0.07) | 0 (0 to 0) | 0.05 (0.03 to 0.08) | 0.39 (0.19 to 0.58) | <0.001 |
| Romania | 0 (0 to 0) | 0.03 (0.02 to 0.04) | 0 (0 to 10) | 0.04 (0.02 to 0.06) | 2.12 (1.37 to 2.88) | <0.001 |
| Russian Federation | 30 (20 to 30) | 0.03 (0.03 to 0.04) | 40 (30 to 50) | 0.06 (0.05 to 0.07) | 2.79 (2.46 to 3.12) | <0.001 |
| Rwanda | 10 (0 to 10) | 0.15 (0.08 to 0.23) | 10 (0 to 10) | 0.1 (0.05 to 0.16) | -1.89 (-2.12 to -1.66) | <0.001 |
| Saint Kitts and Nevis | 0 (0 to 0) | 0.05 (0.03 to 0.07) | 0 (0 to 0) | 0.05 (0.03 to 0.07) | -0.21 (-0.44 to 0.01) | 0.064 |
| Saint Lucia | 0 (0 to 0) | 0.03 (0.02 to 0.04) | 0 (0 to 0) | 0.04 (0.02 to 0.06) | 1.14 (0.87 to 1.42) | <0.001 |
| Saint Vincent and the Grenadines | 0 (0 to 0) | 0.04 (0.03 to 0.06) | 0 (0 to 0) | 0.06 (0.04 to 0.09) | 1.83 (1.66 to 2) | <0.001 |
| Samoa | 0 (0 to 0) | 0.07 (0.04 to 0.13) | 0 (0 to 0) | 0.08 (0.04 to 0.14) | 0.57 (0.47 to 0.66) | <0.001 |
| San Marino | 0 (0 to 0) | 0.02 (0.01 to 0.03) | 0 (0 to 0) | 0.04 (0.02 to 0.07) | 2.72 (2.56 to 2.88) | <0.001 |
| Sao Tome and Principe | 0 (0 to 0) | 0.06 (0.03 to 0.09) | 0 (0 to 0) | 0.06 (0.03 to 0.11) | 0.09 (-0.15 to 0.33) | 0.457 |
| Saudi Arabia | 10 (10 to 20) | 0.1 (0.06 to 0.15) | 30 (20 to 50) | 0.11 (0.06 to 0.19) | 0.72 (0.43 to 1.01) | <0.001 |
| Senegal | 10 (10 to 20) | 0.21 (0.12 to 0.37) | 20 (10 to 20) | 0.2 (0.13 to 0.3) | -0.27 (-0.33 to -0.22) | <0.001 |
| Serbia | 0 (0 to 10) | 0.08 (0.05 to 0.12) | 0 (0 to 0) | 0.06 (0.03 to 0.1) | -1.31 (-1.51 to -1.1) | <0.001 |
| Seychelles | 0 (0 to 0) | 0.07 (0.04 to 0.12) | 0 (0 to 0) | 0.1 (0.06 to 0.16) | 1.62 (1.53 to 1.72) | <0.001 |
| Sierra Leone | 10 (0 to 10) | 0.26 (0.09 to 0.58) | 10 (0 to 10) | 0.18 (0.09 to 0.32) | -1.75 (-1.91 to -1.6) | <0.001 |
| Singapore | 0 (0 to 0) | 0.05 (0.03 to 0.07) | 0 (0 to 0) | 0.03 (0.02 to 0.05) | -2.35 (-2.75 to -1.95) | <0.001 |
| Slovakia | 0 (0 to 0) | 0.06 (0.03 to 0.09) | 0 (0 to 0) | 0.05 (0.03 to 0.09) | -0.27 (-0.31 to -0.22) | <0.001 |
| Slovenia | 0 (0 to 0) | 0.05 (0.03 to 0.08) | 0 (0 to 0) | 0.04 (0.02 to 0.06) | -1.57 (-1.91 to -1.23) | <0.001 |
| Solomon Islands | 0 (0 to 0) | 0.1 (0.04 to 0.18) | 0 (0 to 0) | 0.11 (0.06 to 0.19) | 0.7 (0.54 to 0.86) | <0.001 |
| Somalia | 10 (0 to 20) | 0.18 (0.07 to 0.36) | 10 (10 to 30) | 0.15 (0.06 to 0.31) | -1 (-1.16 to -0.85) | <0.001 |
| South Africa | 70 (60 to 90) | 0.29 (0.23 to 0.35) | 80 (60 to 100) | 0.25 (0.2 to 0.32) | -0.64 (-1 to -0.28) | <0.001 |
| South Sudan | 0 (0 to 10) | 0.1 (0.05 to 0.17) | 10 (0 to 10) | 0.16 (0.08 to 0.26) | 2.16 (1.96 to 2.35) | <0.001 |
| Spain | 10 (10 to 20) | 0.06 (0.04 to 0.08) | 10 (10 to 20) | 0.07 (0.05 to 0.11) | 1.22 (1.04 to 1.4) | <0.001 |
| Sri Lanka | 0 (0 to 10) | 0.03 (0.02 to 0.05) | 0 (0 to 0) | 0.03 (0.01 to 0.04) | -1.24 (-1.69 to -0.78) | <0.001 |
| Sudan | 10 (0 to 10) | 0.05 (0.02 to 0.09) | 20 (10 to 30) | 0.07 (0.04 to 0.12) | 1.99 (1.83 to 2.14) | <0.001 |
| Suriname | 0 (0 to 0) | 0.03 (0.02 to 0.05) | 0 (0 to 0) | 0.06 (0.03 to 0.09) | 2.45 (2.35 to 2.55) | <0.001 |
| Sweden | 0 (0 to 0) | 0.02 (0.02 to 0.03) | 0 (0 to 0) | 0.05 (0.04 to 0.06) | 3.91 (3.65 to 4.18) | <0.001 |
| Switzerland | 0 (0 to 0) | 0.04 (0.03 to 0.07) | 0 (0 to 0) | 0.03 (0.02 to 0.05) | -1.79 (-1.94 to -1.64) | <0.001 |
| Syrian Arab Republic | 10 (0 to 10) | 0.09 (0.05 to 0.13) | 10 (0 to 10) | 0.1 (0.06 to 0.17) | 0.91 (0.08 to 1.75) | 0.032 |
| Taiwan (Province of China) | 30 (20 to 40) | 0.23 (0.15 to 0.34) | 30 (20 to 40) | 0.25 (0.16 to 0.38) | 0.36 (0.02 to 0.7) | 0.038 |
| Tajikistan | 0 (0 to 0) | 0.07 (0.04 to 0.12) | 0 (0 to 10) | 0.06 (0.03 to 0.1) | -1.05 (-1.1 to -1) | <0.001 |
| Thailand | 120 (80 to 190) | 0.34 (0.21 to 0.53) | 110 (60 to 190) | 0.34 (0.18 to 0.59) | 0.02 (-0.29 to 0.33) | 0.889 |
| Timor-Leste | 0 (0 to 0) | 0.04 (0.02 to 0.06) | 0 (0 to 0) | 0.04 (0.02 to 0.07) | 0.36 (0.2 to 0.53) | <0.001 |
| Togo | 0 (0 to 10) | 0.18 (0.1 to 0.3) | 10 (0 to 10) | 0.18 (0.1 to 0.32) | 0.03 (-0.08 to 0.15) | 0.553 |
| Tokelau | 0 (0 to 0) | 0.09 (0.04 to 0.17) | 0 (0 to 0) | 0.12 (0.06 to 0.23) | 1.57 (1.12 to 2.02) | <0.001 |
| Tonga | 0 (0 to 0) | 0.52 (0.31 to 0.81) | 0 (0 to 0) | 0.5 (0.27 to 0.86) | -0.19 (-0.33 to -0.06) | 0.006 |
| Trinidad and Tobago | 0 (0 to 0) | 0.04 (0.03 to 0.06) | 0 (0 to 0) | 0.06 (0.04 to 0.1) | 2.11 (2 to 2.22) | <0.001 |
| Tunisia | 0 (0 to 0) | 0.02 (0.01 to 0.03) | 0 (0 to 0) | 0.04 (0.02 to 0.06) | 2.92 (2.82 to 3.02) | <0.001 |
| Turkey | 10 (10 to 20) | 0.03 (0.02 to 0.04) | 20 (10 to 30) | 0.04 (0.03 to 0.07) | 2.02 (1.76 to 2.29) | <0.001 |
| Turkmenistan | 0 (0 to 0) | 0.08 (0.05 to 0.11) | 0 (0 to 0) | 0.1 (0.06 to 0.16) | 1.47 (1.4 to 1.54) | <0.001 |
| Tuvalu | 0 (0 to 0) | 0.11 (0.06 to 0.19) | 0 (0 to 0) | 0.11 (0.06 to 0.19) | -0.04 (-0.14 to 0.05) | 0.388 |
| Uganda | 20 (10 to 30) | 0.17 (0.11 to 0.26) | 30 (20 to 50) | 0.16 (0.09 to 0.26) | -0.48 (-0.55 to -0.42) | <0.001 |
| Ukraine | 10 (10 to 20) | 0.05 (0.04 to 0.06) | 10 (0 to 10) | 0.04 (0.02 to 0.05) | -1.94 (-2.21 to -1.67) | <0.001 |
| United Arab Emirates | 0 (0 to 0) | 0.1 (0.06 to 0.15) | 10 (10 to 20) | 0.2 (0.11 to 0.32) | 3.43 (3.16 to 3.69) | <0.001 |
| United Kingdom | 10 (10 to 10) | 0.04 (0.03 to 0.04) | 30 (20 to 40) | 0.1 (0.08 to 0.12) | 4.82 (4.66 to 4.97) | <0.001 |
| United Republic of Tanzania | 20 (10 to 30) | 0.12 (0.07 to 0.19) | 30 (20 to 60) | 0.12 (0.06 to 0.2) | 0.09 (0.05 to 0.13) | <0.001 |
| United States of America | 110 (90 to 130) | 0.08 (0.06 to 0.09) | 140 (120 to 160) | 0.09 (0.08 to 0.11) | 0.74 (0.64 to 0.85) | <0.001 |
| United States Virgin Islands | 0 (0 to 0) | 0.05 (0.03 to 0.08) | 0 (0 to 0) | 0.06 (0.03 to 0.11) | 1.05 (0.9 to 1.19) | <0.001 |
| Uruguay | 0 (0 to 0) | 0.01 (0.01 to 0.02) | 0 (0 to 0) | 0.03 (0.02 to 0.05) | 4.95 (4.79 to 5.12) | <0.001 |
| Uzbekistan | 10 (0 to 10) | 0.06 (0.04 to 0.09) | 10 (10 to 20) | 0.08 (0.05 to 0.13) | 1.53 (1.44 to 1.63) | <0.001 |
| Vanuatu | 0 (0 to 0) | 0.08 (0.04 to 0.14) | 0 (0 to 0) | 0.08 (0.04 to 0.15) | 0.33 (0.12 to 0.54) | 0.002 |
| Venezuela (Bolivarian Republic of) | 0 (0 to 10) | 0.03 (0.02 to 0.04) | 10 (10 to 10) | 0.07 (0.04 to 0.1) | 4.27 (4.08 to 4.47) | <0.001 |
| Viet Nam | 70 (40 to 110) | 0.15 (0.09 to 0.26) | 110 (60 to 200) | 0.21 (0.11 to 0.38) | 1.57 (1.46 to 1.68) | <0.001 |
| Yemen | 0 (0 to 0) | 0.02 (0.01 to 0.06) | 0 (0 to 10) | 0.03 (0.01 to 0.05) | 0.2 (0.04 to 0.37) | 0.017 |
| Zambia | 10 (10 to 20) | 0.26 (0.13 to 0.46) | 10 (0 to 30) | 0.11 (0.03 to 0.3) | -4.19 (-4.38 to -3.99) | <0.001 |
| Zimbabwe | 20 (10 to 30) | 0.34 (0.2 to 0.55) | 30 (10 to 50) | 0.34 (0.18 to 0.61) | 0 (-0.1 to 0.1) | 0.953 |

**Abbreviation:** CI: confidence interval; UI: uncertainty interval

**Supplementary Table 4 Death and age-standardized rates of patients with metabolic dysfunction associated steatohepatitis-associated primary liver cancer in young adults in 2000 and 2021 and changes from 2000 to 2021**

| **Country** | 2000 Death (95% UI) | 2000 Age-standardized death rate (95% UI) | 2021 Death (95% UI) | 2021 Age-standardized death rate (95% UI) | 2000 to 2021 Annual Percent Change (95% CI) | ***p*** |
| --- | --- | --- | --- | --- | --- | --- |
| Afghanistan | 10 (0 to 10) | 0.08 (0.05 to 0.14) | 20 (10 to 30) | 0.11 (0.06 to 0.17) | 0.97 (0.66 to 1.28) | <0.001 |
| Albania | 0 (0 to 0) | 0.1 (0.06 to 0.14) | 0 (0 to 0) | 0.1 (0.06 to 0.16) | 0.15 (-0.26 to 0.56) | 0.472 |
| Algeria | 0 (0 to 10) | 0.02 (0.01 to 0.03) | 10 (10 to 20) | 0.04 (0.02 to 0.07) | 2.99 (2.72 to 3.25) | <0.001 |
| American Samoa | 0 (0 to 0) | 0.14 (0.09 to 0.22) | 0 (0 to 0) | 0.21 (0.12 to 0.34) | 2.01 (1.16 to 2.87) | <0.001 |
| Andorra | 0 (0 to 0) | 0.11 (0.06 to 0.19) | 0 (0 to 0) | 0.14 (0.07 to 0.24) | 1.23 (0.97 to 1.5) | <0.001 |
| Angola | 10 (0 to 30) | 0.12 (0.02 to 0.38) | 20 (0 to 40) | 0.11 (0.03 to 0.3) | -0.53 (-0.75 to -0.31) | <0.001 |
| Antigua and Barbuda | 0 (0 to 0) | 0.03 (0.02 to 0.04) | 0 (0 to 0) | 0.03 (0.02 to 0.05) | 0.56 (-0.27 to 1.4) | 0.185 |
| Argentina | 0 (0 to 0) | 0.01 (0.01 to 0.01) | 0 (0 to 10) | 0.02 (0.01 to 0.02) | 3.46 (2.19 to 4.74) | <0.001 |
| Armenia | 0 (0 to 0) | 0.07 (0.05 to 0.11) | 0 (0 to 0) | 0.08 (0.05 to 0.12) | 0.66 (-0.44 to 1.76) | 0.239 |
| Australia | 0 (0 to 10) | 0.05 (0.03 to 0.07) | 10 (10 to 20) | 0.09 (0.06 to 0.13) | 3.26 (2.52 to 4.01) | <0.001 |
| Austria | 0 (0 to 0) | 0.03 (0.02 to 0.04) | 0 (0 to 0) | 0.04 (0.02 to 0.05) | 1.2 (0.45 to 1.96) | 0.002 |
| Azerbaijan | 0 (0 to 10) | 0.08 (0.05 to 0.14) | 10 (0 to 10) | 0.1 (0.05 to 0.19) | 1.04 (0.93 to 1.15) | <0.001 |
| Bahamas | 0 (0 to 0) | 0.06 (0.04 to 0.08) | 0 (0 to 0) | 0.07 (0.04 to 0.1) | 0.85 (0.2 to 1.5) | 0.01 |
| Bahrain | 0 (0 to 0) | 0.06 (0.04 to 0.09) | 0 (0 to 0) | 0.06 (0.03 to 0.09) | -0.72 (-1.48 to 0.05) | 0.068 |
| Bangladesh | 20 (10 to 30) | 0.03 (0.02 to 0.04) | 30 (20 to 50) | 0.04 (0.02 to 0.06) | 1.7 (1.17 to 2.23) | <0.001 |
| Barbados | 0 (0 to 0) | 0.04 (0.03 to 0.06) | 0 (0 to 0) | 0.04 (0.03 to 0.07) | 0.32 (-0.83 to 1.49) | 0.583 |
| Belarus | 0 (0 to 0) | 0.04 (0.02 to 0.05) | 0 (0 to 0) | 0.05 (0.03 to 0.08) | 1.99 (0.78 to 3.21) | 0.001 |
| Belgium | 0 (0 to 0) | 0.03 (0.02 to 0.04) | 0 (0 to 0) | 0.03 (0.02 to 0.05) | 0.72 (0.22 to 1.22) | 0.005 |
| Belize | 0 (0 to 0) | 0.04 (0.02 to 0.05) | 0 (0 to 0) | 0.05 (0.03 to 0.07) | 1.05 (-0.62 to 2.74) | 0.219 |
| Benin | 10 (0 to 20) | 0.33 (0.16 to 0.6) | 10 (10 to 20) | 0.24 (0.13 to 0.4) | -1.6 (-1.88 to -1.32) | <0.001 |
| Bermuda | 0 (0 to 0) | 0.03 (0.02 to 0.04) | 0 (0 to 0) | 0.03 (0.02 to 0.05) | 0.64 (-0.7 to 1.99) | 0.353 |
| Bhutan | 0 (0 to 0) | 0.06 (0.03 to 0.09) | 0 (0 to 0) | 0.07 (0.04 to 0.13) | 1.43 (1.09 to 1.78) | <0.001 |
| Bolivia (Plurinational State of) | 0 (0 to 0) | 0.03 (0.02 to 0.06) | 0 (0 to 0) | 0.04 (0.02 to 0.07) | 0.6 (0.46 to 0.75) | <0.001 |
| Bosnia and Herzegovina | 0 (0 to 0) | 0.08 (0.05 to 0.13) | 0 (0 to 0) | 0.06 (0.03 to 0.1) | -1.46 (-1.93 to -0.98) | <0.001 |
| Botswana | 0 (0 to 0) | 0.15 (0.04 to 0.46) | 0 (0 to 10) | 0.16 (0.06 to 0.38) | 0.33 (-0.61 to 1.28) | 0.491 |
| Brazil | 20 (20 to 20) | 0.02 (0.02 to 0.02) | 30 (30 to 40) | 0.03 (0.02 to 0.03) | 1.08 (0.45 to 1.71) | 0.001 |
| Brunei Darussalam | 0 (0 to 0) | 0.05 (0.03 to 0.09) | 0 (0 to 0) | 0.06 (0.03 to 0.11) | 0.8 (0.35 to 1.26) | 0.001 |
| Bulgaria | 0 (0 to 0) | 0.07 (0.04 to 0.11) | 0 (0 to 0) | 0.06 (0.04 to 0.1) | -0.28 (-1.48 to 0.94) | 0.654 |
| Burkina Faso | 20 (10 to 40) | 0.31 (0.12 to 0.7) | 30 (10 to 70) | 0.3 (0.12 to 0.64) | -0.11 (-0.36 to 0.14) | 0.38 |
| Burundi | 0 (0 to 0) | 0.09 (0.05 to 0.16) | 0 (0 to 10) | 0.06 (0.03 to 0.11) | -1.73 (-1.85 to -1.61) | <0.001 |
| Cote d'Ivoire | 10 (0 to 10) | 0.08 (0.05 to 0.13) | 10 (10 to 20) | 0.07 (0.04 to 0.12) | -0.93 (-1.32 to -0.53) | <0.001 |
| Cabo Verde | 0 (0 to 0) | 0.23 (0.13 to 0.38) | 0 (0 to 0) | 0.28 (0.14 to 0.46) | 0.9 (0.34 to 1.47) | 0.002 |
| Cambodia | 10 (0 to 10) | 0.1 (0.05 to 0.23) | 10 (0 to 20) | 0.09 (0.04 to 0.19) | -0.64 (-0.77 to -0.51) | <0.001 |
| Cameroon | 20 (10 to 40) | 0.34 (0.19 to 0.58) | 40 (20 to 80) | 0.27 (0.14 to 0.49) | -0.98 (-1.22 to -0.75) | <0.001 |
| Canada | 10 (10 to 10) | 0.05 (0.04 to 0.08) | 10 (10 to 20) | 0.07 (0.05 to 0.1) | 1.38 (1.01 to 1.75) | <0.001 |
| Central African Republic | 0 (0 to 10) | 0.14 (0.05 to 0.32) | 0 (0 to 10) | 0.1 (0.04 to 0.22) | -1.56 (-2.14 to -0.98) | <0.001 |
| Chad | 10 (0 to 20) | 0.24 (0.1 to 0.49) | 10 (10 to 30) | 0.18 (0.08 to 0.37) | -1.38 (-1.9 to -0.85) | <0.001 |
| Chile | 0 (0 to 0) | 0.01 (0.01 to 0.02) | 0 (0 to 0) | 0.03 (0.02 to 0.04) | 2.98 (2.61 to 3.36) | <0.001 |
| China | 1170 (910 to 1470) | 0.16 (0.13 to 0.2) | 920 (680 to 1220) | 0.14 (0.1 to 0.18) | -0.73 (-1.22 to -0.23) | 0.004 |
| Colombia | 0 (0 to 10) | 0.02 (0.02 to 0.03) | 10 (10 to 10) | 0.03 (0.02 to 0.05) | 1.5 (0.64 to 2.37) | 0.001 |
| Comoros | 0 (0 to 0) | 0.12 (0.07 to 0.2) | 0 (0 to 0) | 0.14 (0.08 to 0.22) | 0.28 (-2.66 to 3.29) | 0.856 |
| Congo | 0 (0 to 10) | 0.16 (0.06 to 0.38) | 0 (0 to 10) | 0.15 (0.05 to 0.34) | -0.4 (-0.76 to -0.04) | 0.031 |
| Cook Islands | 0 (0 to 0) | 0.22 (0.13 to 0.33) | 0 (0 to 0) | 0.28 (0.15 to 0.47) | 1.31 (0.98 to 1.64) | <0.001 |
| Costa Rica | 0 (0 to 0) | 0.06 (0.04 to 0.09) | 0 (0 to 0) | 0.09 (0.06 to 0.12) | 2.09 (1.29 to 2.9) | <0.001 |
| Croatia | 0 (0 to 0) | 0.03 (0.02 to 0.06) | 0 (0 to 0) | 0.02 (0.01 to 0.04) | -2.18 (-4.78 to 0.49) | 0.109 |
| Cuba | 0 (0 to 0) | 0.03 (0.02 to 0.04) | 0 (0 to 0) | 0.03 (0.02 to 0.05) | 0.48 (-0.14 to 1.11) | 0.129 |
| Cyprus | 0 (0 to 0) | 0.02 (0.01 to 0.04) | 0 (0 to 0) | 0.02 (0.01 to 0.03) | -1.26 (-1.98 to -0.54) | 0.001 |
| Czechia | 0 (0 to 0) | 0.03 (0.02 to 0.05) | 0 (0 to 0) | 0.02 (0.01 to 0.04) | -1.72 (-3.33 to -0.08) | 0.04 |
| Democratic People's Republic of Korea | 20 (10 to 30) | 0.15 (0.07 to 0.27) | 20 (10 to 30) | 0.13 (0.07 to 0.23) | -0.67 (-0.83 to -0.51) | <0.001 |
| Democratic Republic of the Congo | 10 (0 to 30) | 0.05 (0.02 to 0.13) | 20 (10 to 50) | 0.05 (0.02 to 0.11) | -0.37 (-0.68 to -0.05) | 0.023 |
| Denmark | 0 (0 to 0) | 0.02 (0.01 to 0.03) | 0 (0 to 0) | 0.01 (0.01 to 0.02) | -2.32 (-3.36 to -1.26) | <0.001 |
| Djibouti | 0 (0 to 0) | 0.09 (0.05 to 0.15) | 0 (0 to 0) | 0.11 (0.05 to 0.18) | 0.89 (0.76 to 1.02) | <0.001 |
| Dominica | 0 (0 to 0) | 0.04 (0.02 to 0.06) | 0 (0 to 0) | 0.05 (0.03 to 0.08) | 1.17 (0.78 to 1.56) | <0.001 |
| Dominican Republic | 0 (0 to 0) | 0.02 (0.01 to 0.03) | 0 (0 to 0) | 0.04 (0.02 to 0.06) | 2.97 (2.37 to 3.56) | <0.001 |
| Ecuador | 0 (0 to 0) | 0.03 (0.02 to 0.04) | 0 (0 to 10) | 0.04 (0.03 to 0.06) | 1.98 (0.93 to 3.04) | <0.001 |
| Egypt | 70 (40 to 110) | 0.21 (0.12 to 0.32) | 160 (100 to 240) | 0.3 (0.18 to 0.44) | 1.64 (1.46 to 1.81) | <0.001 |
| El Salvador | 0 (0 to 0) | 0.02 (0.01 to 0.03) | 0 (0 to 0) | 0.03 (0.02 to 0.05) | 2.31 (0.86 to 3.79) | 0.002 |
| Equatorial Guinea | 0 (0 to 0) | 0.05 (0.02 to 0.09) | 0 (0 to 0) | 0.09 (0.04 to 0.16) | 2.98 (2.31 to 3.66) | <0.001 |
| Eritrea | 0 (0 to 0) | 0.09 (0.05 to 0.16) | 0 (0 to 10) | 0.09 (0.04 to 0.15) | -0.18 (-0.5 to 0.14) | 0.264 |
| Estonia | 0 (0 to 0) | 0.06 (0.04 to 0.08) | 0 (0 to 0) | 0.05 (0.03 to 0.07) | -0.21 (-0.69 to 0.28) | 0.398 |
| Eswatini | 0 (0 to 0) | 0.53 (0.27 to 0.92) | 0 (0 to 10) | 0.58 (0.18 to 1.39) | 0.29 (-0.04 to 0.62) | 0.082 |
| Ethiopia | 20 (10 to 30) | 0.06 (0.04 to 0.08) | 30 (20 to 40) | 0.05 (0.03 to 0.07) | -1.15 (-1.39 to -0.91) | <0.001 |
| Fiji | 0 (0 to 0) | 0.07 (0.04 to 0.1) | 0 (0 to 0) | 0.11 (0.06 to 0.18) | 2.47 (1.73 to 3.22) | <0.001 |
| Finland | 0 (0 to 0) | 0.03 (0.02 to 0.05) | 0 (0 to 0) | 0.03 (0.02 to 0.04) | -0.7 (-1.91 to 0.52) | 0.26 |
| France | 10 (10 to 20) | 0.05 (0.03 to 0.07) | 10 (10 to 20) | 0.05 (0.03 to 0.07) | 0.17 (-0.21 to 0.56) | 0.374 |
| Gabon | 0 (0 to 0) | 0.18 (0.08 to 0.37) | 0 (0 to 0) | 0.18 (0.09 to 0.33) | 0.04 (-0.39 to 0.47) | 0.85 |
| Gambia | 0 (0 to 0) | 0.5 (0.3 to 0.77) | 10 (0 to 10) | 0.61 (0.33 to 1.09) | 1.32 (-0.2 to 2.86) | 0.089 |
| Georgia | 0 (0 to 0) | 0.05 (0.03 to 0.08) | 0 (0 to 0) | 0.06 (0.04 to 0.08) | -0.19 (-2.38 to 2.05) | 0.865 |
| Germany | 10 (10 to 20) | 0.03 (0.02 to 0.04) | 10 (10 to 20) | 0.03 (0.02 to 0.05) | 0.72 (0.36 to 1.09) | <0.001 |
| Ghana | 30 (20 to 40) | 0.28 (0.17 to 0.49) | 30 (20 to 60) | 0.18 (0.1 to 0.31) | -2.14 (-2.29 to -1.99) | <0.001 |
| Greece | 0 (0 to 0) | 0.02 (0.02 to 0.03) | 0 (0 to 0) | 0.04 (0.03 to 0.06) | 2.76 (1.98 to 3.54) | <0.001 |
| Greenland | 0 (0 to 0) | 0.1 (0.06 to 0.16) | 0 (0 to 0) | 0.08 (0.04 to 0.13) | -1.07 (-1.36 to -0.77) | <0.001 |
| Grenada | 0 (0 to 0) | 0.03 (0.02 to 0.04) | 0 (0 to 0) | 0.05 (0.03 to 0.08) | 2.81 (0.64 to 5.03) | 0.011 |
| Guam | 0 (0 to 0) | 0.08 (0.05 to 0.12) | 0 (0 to 0) | 0.15 (0.09 to 0.24) | 3.13 (2.57 to 3.69) | <0.001 |
| Guatemala | 0 (0 to 0) | 0.05 (0.03 to 0.07) | 10 (0 to 10) | 0.08 (0.05 to 0.11) | 2.39 (1.04 to 3.76) | <0.001 |
| Guinea | 10 (10 to 20) | 0.42 (0.26 to 0.68) | 20 (10 to 40) | 0.39 (0.21 to 0.64) | -0.44 (-0.71 to -0.18) | 0.001 |
| Guinea-Bissau | 0 (0 to 0) | 0.46 (0.2 to 0.82) | 0 (0 to 10) | 0.35 (0.19 to 0.6) | -1.24 (-1.75 to -0.74) | <0.001 |
| Guyana | 0 (0 to 0) | 0.04 (0.03 to 0.06) | 0 (0 to 0) | 0.05 (0.03 to 0.07) | 0.81 (0.02 to 1.6) | 0.045 |
| Haiti | 0 (0 to 0) | 0.02 (0.01 to 0.04) | 0 (0 to 0) | 0.02 (0.01 to 0.04) | -0.31 (-0.63 to 0.01) | 0.058 |
| Honduras | 0 (0 to 0) | 0.03 (0.02 to 0.05) | 0 (0 to 0) | 0.04 (0.02 to 0.07) | 0.5 (0.31 to 0.69) | <0.001 |
| Hungary | 0 (0 to 0) | 0.04 (0.02 to 0.06) | 0 (0 to 0) | 0.02 (0.01 to 0.03) | -2.97 (-3.67 to -2.26) | <0.001 |
| Iceland | 0 (0 to 0) | 0.02 (0.01 to 0.03) | 0 (0 to 0) | 0.03 (0.02 to 0.04) | 1.11 (-0.29 to 2.52) | 0.121 |
| India | 260 (210 to 320) | 0.05 (0.04 to 0.06) | 490 (380 to 610) | 0.06 (0.05 to 0.08) | 1.09 (0.82 to 1.35) | <0.001 |
| Indonesia | 90 (60 to 130) | 0.08 (0.05 to 0.11) | 160 (100 to 260) | 0.11 (0.06 to 0.17) | 1.54 (1.4 to 1.68) | <0.001 |
| Iran (Islamic Republic of) | 10 (10 to 20) | 0.03 (0.03 to 0.04) | 30 (20 to 30) | 0.05 (0.04 to 0.07) | 2.26 (1.52 to 3) | <0.001 |
| Iraq | 10 (0 to 10) | 0.05 (0.03 to 0.08) | 20 (10 to 30) | 0.07 (0.04 to 0.12) | 1.89 (1.65 to 2.13) | <0.001 |
| Ireland | 0 (0 to 0) | 0.02 (0.01 to 0.03) | 0 (0 to 0) | 0.03 (0.02 to 0.04) | 1.07 (0.18 to 1.97) | 0.019 |
| Israel | 0 (0 to 0) | 0.02 (0.01 to 0.03) | 0 (0 to 0) | 0.02 (0.01 to 0.03) | 0.09 (-1.07 to 1.26) | 0.881 |
| Italy | 10 (10 to 10) | 0.03 (0.03 to 0.04) | 10 (10 to 10) | 0.03 (0.02 to 0.03) | -0.68 (-2.16 to 0.83) | 0.375 |
| Jamaica | 0 (0 to 0) | 0.02 (0.01 to 0.03) | 0 (0 to 0) | 0.03 (0.02 to 0.05) | 1.39 (-0.95 to 3.79) | 0.247 |
| Japan | 40 (30 to 50) | 0.07 (0.05 to 0.08) | 10 (10 to 20) | 0.03 (0.02 to 0.03) | -4.17 (-4.72 to -3.62) | <0.001 |
| Jordan | 0 (0 to 0) | 0.02 (0.01 to 0.03) | 0 (0 to 0) | 0.02 (0.01 to 0.04) | 0.49 (0.05 to 0.94) | 0.029 |
| Kazakhstan | 10 (10 to 20) | 0.16 (0.11 to 0.23) | 10 (0 to 10) | 0.08 (0.05 to 0.11) | -3.77 (-5.3 to -2.21) | <0.001 |
| Kenya | 10 (10 to 20) | 0.08 (0.05 to 0.12) | 30 (20 to 50) | 0.12 (0.08 to 0.17) | 2.25 (2.03 to 2.48) | <0.001 |
| Kiribati | 0 (0 to 0) | 0.18 (0.11 to 0.28) | 0 (0 to 0) | 0.16 (0.09 to 0.27) | -0.64 (-0.94 to -0.35) | <0.001 |
| Kuwait | 0 (0 to 0) | 0.03 (0.02 to 0.04) | 0 (0 to 0) | 0.02 (0.01 to 0.03) | -3.17 (-5.1 to -1.21) | 0.002 |
| Kyrgyzstan | 0 (0 to 0) | 0.04 (0.02 to 0.06) | 0 (0 to 0) | 0.03 (0.02 to 0.05) | -0.98 (-1.86 to -0.1) | 0.03 |
| Lao People's Democratic Republic | 0 (0 to 0) | 0.11 (0.07 to 0.2) | 0 (0 to 10) | 0.1 (0.05 to 0.16) | -0.93 (-1.02 to -0.84) | <0.001 |
| Latvia | 0 (0 to 0) | 0.04 (0.03 to 0.07) | 0 (0 to 0) | 0.05 (0.03 to 0.08) | 0.32 (-0.09 to 0.72) | 0.121 |
| Lebanon | 0 (0 to 0) | 0.03 (0.02 to 0.04) | 0 (0 to 0) | 0.03 (0.02 to 0.05) | 0.5 (0.01 to 0.98) | 0.044 |
| Lesotho | 0 (0 to 0) | 0.26 (0.11 to 0.58) | 0 (0 to 10) | 0.4 (0.13 to 1.07) | 2.23 (1.97 to 2.48) | <0.001 |
| Liberia | 0 (0 to 10) | 0.29 (0.13 to 0.57) | 10 (10 to 20) | 0.37 (0.2 to 0.64) | 1.18 (0.66 to 1.7) | <0.001 |
| Libya | 0 (0 to 0) | 0.08 (0.05 to 0.12) | 10 (0 to 10) | 0.18 (0.1 to 0.31) | 4.07 (3.82 to 4.33) | <0.001 |
| Lithuania | 0 (0 to 0) | 0.03 (0.02 to 0.05) | 0 (0 to 0) | 0.05 (0.03 to 0.07) | 1.75 (0.64 to 2.86) | 0.002 |
| Luxembourg | 0 (0 to 0) | 0.03 (0.02 to 0.04) | 0 (0 to 0) | 0.03 (0.02 to 0.04) | -0.54 (-1.21 to 0.14) | 0.119 |
| Madagascar | 10 (0 to 10) | 0.07 (0.04 to 0.12) | 10 (10 to 20) | 0.07 (0.04 to 0.12) | 0.2 (-0.03 to 0.43) | 0.088 |
| Malawi | 10 (0 to 10) | 0.11 (0.07 to 0.2) | 10 (10 to 20) | 0.1 (0.06 to 0.17) | -0.5 (-0.75 to -0.25) | <0.001 |
| Malaysia | 10 (10 to 10) | 0.07 (0.04 to 0.1) | 10 (10 to 20) | 0.08 (0.05 to 0.13) | 0.95 (0.32 to 1.57) | 0.003 |
| Maldives | 0 (0 to 0) | 0.03 (0.02 to 0.06) | 0 (0 to 0) | 0.04 (0.02 to 0.07) | 0.33 (-0.14 to 0.8) | 0.17 |
| Mali | 20 (10 to 30) | 0.34 (0.21 to 0.55) | 40 (20 to 60) | 0.36 (0.21 to 0.59) | 0.31 (0.11 to 0.51) | 0.002 |
| Malta | 0 (0 to 0) | 0.02 (0.01 to 0.03) | 0 (0 to 0) | 0.02 (0.01 to 0.03) | 0.94 (0.03 to 1.87) | 0.043 |
| Marshall Islands | 0 (0 to 0) | 0.07 (0.04 to 0.12) | 0 (0 to 0) | 0.09 (0.05 to 0.16) | 1.21 (1.1 to 1.33) | <0.001 |
| Mauritania | 10 (0 to 10) | 0.5 (0.15 to 1.11) | 10 (0 to 10) | 0.36 (0.17 to 0.63) | -1.59 (-1.85 to -1.33) | <0.001 |
| Mauritius | 0 (0 to 0) | 0.02 (0.01 to 0.03) | 0 (0 to 0) | 0.01 (0.01 to 0.02) | -2.71 (-3.74 to -1.67) | <0.001 |
| Mexico | 20 (10 to 20) | 0.03 (0.03 to 0.04) | 30 (30 to 40) | 0.05 (0.04 to 0.06) | 2.08 (1.84 to 2.32) | <0.001 |
| Micronesia (Federated States of) | 0 (0 to 0) | 0.13 (0.07 to 0.22) | 0 (0 to 0) | 0.13 (0.06 to 0.22) | -0.04 (-0.32 to 0.24) | 0.768 |
| Monaco | 0 (0 to 0) | 0.08 (0.05 to 0.14) | 0 (0 to 0) | 0.11 (0.06 to 0.18) | 1.25 (1.01 to 1.49) | <0.001 |
| Mongolia | 10 (0 to 10) | 0.6 (0.33 to 1.03) | 10 (10 to 20) | 0.72 (0.43 to 1.21) | 0.93 (0.53 to 1.34) | <0.001 |
| Montenegro | 0 (0 to 0) | 0.09 (0.06 to 0.14) | 0 (0 to 0) | 0.09 (0.05 to 0.14) | 0.1 (-0.29 to 0.5) | 0.615 |
| Morocco | 0 (0 to 0) | 0.01 (0 to 0.01) | 0 (0 to 0) | 0.01 (0.01 to 0.02) | 2.58 (2.46 to 2.7) | <0.001 |
| Mozambique | 10 (10 to 20) | 0.18 (0.1 to 0.29) | 30 (10 to 60) | 0.21 (0.1 to 0.42) | 0.87 (0.66 to 1.08) | <0.001 |
| Myanmar | 10 (0 to 30) | 0.05 (0.02 to 0.11) | 20 (10 to 30) | 0.06 (0.02 to 0.12) | 0.2 (-0.04 to 0.44) | 0.105 |
| Namibia | 0 (0 to 0) | 0.06 (0.03 to 0.1) | 0 (0 to 0) | 0.06 (0.03 to 0.1) | -0.06 (-0.6 to 0.47) | 0.818 |
| Nauru | 0 (0 to 0) | 0.17 (0.09 to 0.29) | 0 (0 to 0) | 0.16 (0.08 to 0.29) | -0.11 (-0.33 to 0.12) | 0.361 |
| Nepal | 0 (0 to 0) | 0.03 (0.02 to 0.04) | 10 (10 to 20) | 0.05 (0.03 to 0.09) | 3.3 (2.95 to 3.64) | <0.001 |
| Netherlands | 0 (0 to 0) | 0.02 (0.01 to 0.03) | 0 (0 to 0) | 0.03 (0.02 to 0.04) | 0.75 (-0.14 to 1.65) | 0.1 |
| New Zealand | 0 (0 to 0) | 0.06 (0.05 to 0.08) | 0 (0 to 0) | 0.1 (0.08 to 0.12) | 2.11 (0.56 to 3.69) | 0.008 |
| Nicaragua | 0 (0 to 0) | 0.03 (0.02 to 0.04) | 0 (0 to 0) | 0.04 (0.03 to 0.07) | 2 (1.61 to 2.4) | <0.001 |
| Niger | 10 (0 to 20) | 0.22 (0.1 to 0.47) | 10 (10 to 30) | 0.13 (0.07 to 0.25) | -2.42 (-2.77 to -2.07) | <0.001 |
| Nigeria | 50 (20 to 90) | 0.08 (0.04 to 0.16) | 70 (40 to 130) | 0.07 (0.04 to 0.12) | -0.95 (-1.21 to -0.68) | <0.001 |
| Niue | 0 (0 to 0) | 0.13 (0.07 to 0.21) | 0 (0 to 0) | 0.13 (0.07 to 0.23) | 0.17 (-0.07 to 0.4) | 0.156 |
| North Macedonia | 0 (0 to 0) | 0.09 (0.06 to 0.15) | 0 (0 to 0) | 0.08 (0.05 to 0.14) | -0.5 (-0.76 to -0.24) | <0.001 |
| Northern Mariana Islands | 0 (0 to 0) | 0.11 (0.06 to 0.18) | 0 (0 to 0) | 0.13 (0.07 to 0.23) | 0.36 (-0.79 to 1.52) | 0.543 |
| Norway | 0 (0 to 0) | 0.02 (0.02 to 0.03) | 0 (0 to 0) | 0.04 (0.03 to 0.05) | 2.82 (1.82 to 3.83) | <0.001 |
| Oman | 0 (0 to 0) | 0.04 (0.02 to 0.06) | 0 (0 to 0) | 0.05 (0.03 to 0.09) | 1.6 (0.56 to 2.66) | 0.002 |
| Pakistan | 50 (30 to 60) | 0.07 (0.05 to 0.09) | 100 (70 to 130) | 0.08 (0.06 to 0.11) | 0.7 (0.46 to 0.95) | <0.001 |
| Palau | 0 (0 to 0) | 0.21 (0.1 to 0.37) | 0 (0 to 0) | 0.28 (0.14 to 0.52) | 1.31 (0.86 to 1.76) | <0.001 |
| Palestine | 0 (0 to 0) | 0.05 (0.03 to 0.08) | 0 (0 to 0) | 0.05 (0.03 to 0.08) | 0 (-0.45 to 0.46) | 0.985 |
| Panama | 0 (0 to 0) | 0.02 (0.02 to 0.03) | 0 (0 to 0) | 0.05 (0.03 to 0.07) | 3.48 (3 to 3.95) | <0.001 |
| Papua New Guinea | 0 (0 to 0) | 0.05 (0.02 to 0.11) | 0 (0 to 0) | 0.04 (0.02 to 0.09) | -1.07 (-1.39 to -0.74) | <0.001 |
| Paraguay | 0 (0 to 0) | 0.02 (0.01 to 0.03) | 0 (0 to 0) | 0.04 (0.02 to 0.06) | 2.53 (2.27 to 2.78) | <0.001 |
| Peru | 0 (0 to 0) | 0.02 (0.01 to 0.04) | 10 (0 to 10) | 0.03 (0.02 to 0.05) | 1.87 (0.19 to 3.59) | 0.029 |
| Philippines | 50 (40 to 60) | 0.12 (0.09 to 0.15) | 60 (50 to 80) | 0.11 (0.08 to 0.14) | -0.48 (-0.76 to -0.2) | 0.001 |
| Poland | 0 (0 to 0) | 0.01 (0.01 to 0.01) | 0 (0 to 10) | 0.03 (0.02 to 0.03) | 5.82 (3.5 to 8.19) | <0.001 |
| Portugal | 0 (0 to 0) | 0.03 (0.02 to 0.05) | 0 (0 to 0) | 0.05 (0.03 to 0.07) | 1.43 (0.12 to 2.76) | 0.032 |
| Puerto Rico | 0 (0 to 0) | 0.05 (0.03 to 0.07) | 0 (0 to 0) | 0.07 (0.04 to 0.11) | 1.89 (-0.07 to 3.89) | 0.059 |
| Qatar | 0 (0 to 0) | 0.14 (0.08 to 0.22) | 0 (0 to 0) | 0.11 (0.06 to 0.18) | -1.18 (-2.41 to 0.06) | 0.063 |
| Republic of Korea | 50 (30 to 80) | 0.2 (0.12 to 0.3) | 30 (20 to 50) | 0.11 (0.07 to 0.19) | -2.53 (-2.83 to -2.23) | <0.001 |
| Republic of Moldova | 0 (0 to 0) | 0.05 (0.03 to 0.07) | 0 (0 to 0) | 0.05 (0.03 to 0.07) | 0.56 (-0.94 to 2.08) | 0.465 |
| Romania | 0 (0 to 0) | 0.02 (0.01 to 0.04) | 0 (0 to 0) | 0.04 (0.02 to 0.06) | 2.57 (2.12 to 3.02) | <0.001 |
| Russian Federation | 20 (20 to 30) | 0.03 (0.03 to 0.04) | 40 (30 to 40) | 0.05 (0.04 to 0.06) | 2.58 (0.15 to 5.07) | 0.038 |
| Rwanda | 10 (0 to 10) | 0.14 (0.08 to 0.22) | 10 (0 to 10) | 0.09 (0.05 to 0.16) | -1.89 (-2.01 to -1.76) | <0.001 |
| Saint Kitts and Nevis | 0 (0 to 0) | 0.04 (0.03 to 0.06) | 0 (0 to 0) | 0.04 (0.02 to 0.06) | -0.22 (-1.11 to 0.68) | 0.638 |
| Saint Lucia | 0 (0 to 0) | 0.03 (0.02 to 0.04) | 0 (0 to 0) | 0.03 (0.02 to 0.05) | 1.32 (0.18 to 2.48) | 0.024 |
| Saint Vincent and the Grenadines | 0 (0 to 0) | 0.04 (0.02 to 0.06) | 0 (0 to 0) | 0.06 (0.04 to 0.08) | 1.71 (0.74 to 2.7) | 0.001 |
| Samoa | 0 (0 to 0) | 0.07 (0.03 to 0.12) | 0 (0 to 0) | 0.07 (0.04 to 0.13) | 0.47 (-0.01 to 0.96) | 0.054 |
| San Marino | 0 (0 to 0) | 0.01 (0.01 to 0.02) | 0 (0 to 0) | 0.02 (0.01 to 0.05) | 2.65 (2.48 to 2.82) | <0.001 |
| Sao Tome and Principe | 0 (0 to 0) | 0.05 (0.03 to 0.08) | 0 (0 to 0) | 0.05 (0.02 to 0.1) | -0.24 (-1.18 to 0.7) | 0.613 |
| Saudi Arabia | 10 (10 to 20) | 0.09 (0.05 to 0.14) | 20 (10 to 40) | 0.1 (0.05 to 0.16) | 0.44 (0.2 to 0.69) | <0.001 |
| Senegal | 10 (10 to 20) | 0.2 (0.11 to 0.35) | 10 (10 to 20) | 0.19 (0.12 to 0.28) | -0.85 (-1.51 to -0.19) | 0.012 |
| Serbia | 0 (0 to 10) | 0.07 (0.04 to 0.11) | 0 (0 to 0) | 0.05 (0.03 to 0.09) | -1.41 (-1.76 to -1.06) | <0.001 |
| Seychelles | 0 (0 to 0) | 0.07 (0.04 to 0.11) | 0 (0 to 0) | 0.09 (0.05 to 0.14) | 1.6 (1.26 to 1.94) | <0.001 |
| Sierra Leone | 0 (0 to 10) | 0.24 (0.09 to 0.54) | 10 (0 to 10) | 0.17 (0.09 to 0.29) | -1.74 (-2.06 to -1.42) | <0.001 |
| Singapore | 0 (0 to 0) | 0.04 (0.02 to 0.06) | 0 (0 to 0) | 0.02 (0.01 to 0.03) | -3.57 (-4.67 to -2.47) | <0.001 |
| Slovakia | 0 (0 to 0) | 0.05 (0.03 to 0.08) | 0 (0 to 0) | 0.05 (0.02 to 0.08) | -0.38 (-0.94 to 0.18) | 0.181 |
| Slovenia | 0 (0 to 0) | 0.05 (0.03 to 0.07) | 0 (0 to 0) | 0.03 (0.02 to 0.05) | -2.09 (-3.08 to -1.09) | <0.001 |
| Solomon Islands | 0 (0 to 0) | 0.09 (0.04 to 0.17) | 0 (0 to 0) | 0.1 (0.05 to 0.17) | 0.7 (0.4 to 0.99) | <0.001 |
| Somalia | 10 (0 to 20) | 0.17 (0.07 to 0.33) | 10 (10 to 30) | 0.14 (0.06 to 0.29) | -1.02 (-1.12 to -0.92) | <0.001 |
| South Africa | 60 (50 to 80) | 0.27 (0.21 to 0.32) | 70 (60 to 90) | 0.23 (0.18 to 0.29) | -0.64 (-1.28 to 0.01) | 0.053 |
| South Sudan | 0 (0 to 10) | 0.09 (0.05 to 0.16) | 10 (0 to 10) | 0.15 (0.08 to 0.25) | 2.18 (1.45 to 2.91) | <0.001 |
| Spain | 10 (10 to 10) | 0.04 (0.03 to 0.06) | 10 (10 to 10) | 0.04 (0.03 to 0.07) | 0.28 (-0.62 to 1.19) | 0.548 |
| Sri Lanka | 0 (0 to 0) | 0.03 (0.02 to 0.05) | 0 (0 to 0) | 0.02 (0.01 to 0.04) | -1.52 (-2.13 to -0.91) | <0.001 |
| Sudan | 10 (0 to 10) | 0.05 (0.02 to 0.08) | 10 (10 to 30) | 0.07 (0.03 to 0.11) | 1.88 (1.65 to 2.11) | <0.001 |
| Suriname | 0 (0 to 0) | 0.03 (0.02 to 0.05) | 0 (0 to 0) | 0.05 (0.03 to 0.09) | 2.54 (1.82 to 3.26) | <0.001 |
| Sweden | 0 (0 to 0) | 0.02 (0.01 to 0.02) | 0 (0 to 0) | 0.04 (0.03 to 0.04) | 3.33 (2.29 to 4.37) | <0.001 |
| Switzerland | 0 (0 to 0) | 0.03 (0.02 to 0.04) | 0 (0 to 0) | 0.02 (0.01 to 0.03) | -2.31 (-3.03 to -1.59) | <0.001 |
| Syrian Arab Republic | 10 (0 to 10) | 0.08 (0.05 to 0.12) | 10 (0 to 10) | 0.09 (0.05 to 0.14) | 0.8 (0.22 to 1.38) | 0.007 |
| Taiwan (Province of China) | 20 (10 to 30) | 0.16 (0.1 to 0.24) | 20 (10 to 30) | 0.17 (0.11 to 0.25) | -0.75 (-1.69 to 0.21) | 0.125 |
| Tajikistan | 0 (0 to 0) | 0.07 (0.04 to 0.11) | 0 (0 to 0) | 0.05 (0.03 to 0.09) | -1.11 (-1.4 to -0.81) | <0.001 |
| Thailand | 110 (70 to 170) | 0.31 (0.19 to 0.47) | 90 (50 to 160) | 0.29 (0.15 to 0.5) | -0.31 (-0.78 to 0.17) | 0.209 |
| Timor-Leste | 0 (0 to 0) | 0.03 (0.02 to 0.06) | 0 (0 to 0) | 0.04 (0.02 to 0.07) | 0.31 (-0.47 to 1.08) | 0.439 |
| Togo | 0 (0 to 10) | 0.17 (0.09 to 0.28) | 10 (0 to 10) | 0.17 (0.09 to 0.3) | -0.02 (-0.37 to 0.32) | 0.893 |
| Tokelau | 0 (0 to 0) | 0.08 (0.04 to 0.16) | 0 (0 to 0) | 0.11 (0.06 to 0.21) | 1.47 (0.99 to 1.96) | <0.001 |
| Tonga | 0 (0 to 0) | 0.47 (0.29 to 0.73) | 0 (0 to 0) | 0.45 (0.24 to 0.79) | -0.24 (-0.75 to 0.28) | 0.365 |
| Trinidad and Tobago | 0 (0 to 0) | 0.04 (0.02 to 0.05) | 0 (0 to 0) | 0.06 (0.03 to 0.09) | 2.12 (0.41 to 3.85) | 0.015 |
| Tunisia | 0 (0 to 0) | 0.02 (0.01 to 0.03) | 0 (0 to 0) | 0.03 (0.02 to 0.05) | 2.68 (2.49 to 2.88) | <0.001 |
| Turkey | 10 (10 to 10) | 0.03 (0.02 to 0.04) | 20 (10 to 30) | 0.04 (0.02 to 0.06) | 1.68 (1.12 to 2.24) | <0.001 |
| Turkmenistan | 0 (0 to 0) | 0.07 (0.05 to 0.1) | 0 (0 to 0) | 0.1 (0.06 to 0.14) | 1.72 (1.51 to 1.93) | <0.001 |
| Tuvalu | 0 (0 to 0) | 0.1 (0.06 to 0.17) | 0 (0 to 0) | 0.1 (0.06 to 0.17) | -0.13 (-0.23 to -0.02) | 0.023 |
| Uganda | 20 (10 to 30) | 0.16 (0.1 to 0.24) | 30 (20 to 50) | 0.15 (0.08 to 0.24) | -0.61 (-0.98 to -0.23) | 0.002 |
| Ukraine | 10 (10 to 20) | 0.05 (0.04 to 0.06) | 10 (0 to 10) | 0.03 (0.02 to 0.04) | -1.85 (-3.97 to 0.31) | 0.092 |
| United Arab Emirates | 0 (0 to 0) | 0.09 (0.05 to 0.14) | 10 (10 to 20) | 0.17 (0.1 to 0.28) | 3.17 (2.53 to 3.81) | <0.001 |
| United Kingdom | 10 (10 to 10) | 0.03 (0.02 to 0.03) | 20 (10 to 20) | 0.06 (0.05 to 0.07) | 4.05 (3.8 to 4.31) | <0.001 |
| United Republic of Tanzania | 20 (10 to 30) | 0.11 (0.06 to 0.18) | 30 (20 to 50) | 0.11 (0.06 to 0.18) | 0.07 (-0.03 to 0.17) | 0.151 |
| United States of America | 70 (60 to 80) | 0.05 (0.04 to 0.06) | 80 (60 to 90) | 0.05 (0.04 to 0.06) | 0.4 (-0.21 to 1) | 0.197 |
| United States Virgin Islands | 0 (0 to 0) | 0.04 (0.03 to 0.07) | 0 (0 to 0) | 0.05 (0.03 to 0.09) | 0.92 (0.66 to 1.19) | <0.001 |
| Uruguay | 0 (0 to 0) | 0.01 (0.01 to 0.02) | 0 (0 to 0) | 0.03 (0.02 to 0.04) | 4.41 (3.55 to 5.27) | <0.001 |
| Uzbekistan | 10 (0 to 10) | 0.05 (0.03 to 0.08) | 10 (10 to 20) | 0.07 (0.04 to 0.12) | 1.58 (0.7 to 2.46) | <0.001 |
| Vanuatu | 0 (0 to 0) | 0.07 (0.04 to 0.13) | 0 (0 to 0) | 0.08 (0.04 to 0.13) | 0.23 (-0.14 to 0.59) | 0.22 |
| Venezuela (Bolivarian Republic of) | 0 (0 to 0) | 0.03 (0.02 to 0.04) | 10 (10 to 10) | 0.06 (0.04 to 0.09) | 4.04 (3.33 to 4.76) | <0.001 |
| Viet Nam | 60 (30 to 100) | 0.14 (0.08 to 0.23) | 90 (50 to 170) | 0.18 (0.1 to 0.33) | 1.29 (1 to 1.57) | <0.001 |
| Yemen | 0 (0 to 0) | 0.02 (0.01 to 0.05) | 0 (0 to 10) | 0.02 (0.01 to 0.05) | 0.2 (-0.88 to 1.29) | 0.72 |
| Zambia | 10 (10 to 20) | 0.24 (0.12 to 0.43) | 10 (0 to 30) | 0.1 (0.03 to 0.29) | -4.14 (-4.69 to -3.59) | <0.001 |
| Zimbabwe | 20 (10 to 30) | 0.32 (0.18 to 0.51) | 20 (10 to 40) | 0.32 (0.17 to 0.56) | 0.01 (-0.45 to 0.46) | 0.973 |

**Abbreviation:** CI: confidence interval; UI: uncertainty interval

**Supplementary Table 5 Age-standardized incidence rates, death rates, disability-adjusted life years of patients with metabolic dysfunction associated steatohepatitis-associated primary liver cancer in young and older adults in 2021 and changes from 2000 to 2021 by age group**

|  | 2021 ASIR (95% UI) | 2000 to 2021 APC (95% CI) | ***p*** | 2021 ASDR (95% UI) | 2000 to 2021 APC (95% CI) | ***p*** | 2021 ASDALYs (95% UI) | 2000 to 2021 APC (95% CI) | ***p*** |
| --- | --- | --- | --- | --- | --- | --- | --- | --- | --- |
| 15-19 years | 0.03 (0.02 to 0.03) | 0.59 (0.57 to 0.62) | <0.001 | 0.02 (0.02 to 0.03) | 0.46 (0.39 to 0.53) | <0.001 | 1.72 (1.21 to 2.35) | 0.46 (0.39 to 0.53) | <0.001 |
| 20-24 years | 0.03 (0.02 to 0.04) | 0.41 (0.3 to 0.52) | <0.001 | 0.03 (0.02 to 0.04) | 0.22 (0.04 to 0.4) | 0.016 | 1.84 (1.34 to 2.51) | 0.22 (0.04 to 0.4) | 0.016 |
| 25-29 years | 0.04 (0.03 to 0.06) | 0.18 (0.06 to 0.29) | 0.002 | 0.04 (0.02 to 0.05) | -0.03 (-0.18 to 0.12) | 0.705 | 2.22 (1.46 to 3.38) | -0.03 (-0.18 to 0.13) | 0.732 |
| 30-34 years | 0.06 (0.05 to 0.09) | -0.22 (-0.35 to -0.08) | 0.001 | 0.05 (0.04 to 0.08) | -0.57 (-0.79 to -0.35) | <0.001 | 3.12 (2.19 to 4.38) | -0.57 (-0.79 to -0.35) | <0.001 |
| 35-39 years | 0.11 (0.08 to 0.16) | -0.21 (-0.28 to -0.14) | <0.001 | 0.09 (0.06 to 0.12) | -0.61 (-1.14 to -0.07) | 0.026 | 4.68 (3.12 to 6.59) | -0.61 (-1.14 to -0.08) | 0.025 |
| 40-44 years | 0.2 (0.13 to 0.27) | -0.09 (-0.12 to -0.05) | <0.001 | 0.16 (0.11 to 0.22) | -0.4 (-0.59 to -0.21) | <0.001 | 7.63 (5.23 to 10.75) | -0.35 (-0.66 to -0.05) | 0.024 |
| 45-49 years | 0.36 (0.26 to 0.5) | -0.23 (-0.53 to 0.07) | 0.127 | 0.3 (0.21 to 0.41) | -0.57 (-0.75 to -0.39) | <0.001 | 12.93 (8.95 to 17.89) | -0.57 (-0.75 to -0.38) | <0.001 |
| >55 years | 2.36 (1.86 to 2.94) | 0.72 (0.63 to 0.8) | <0.001 | 2.35 (1.85 to 2.91) | 0.57 (0.43 to 0.71) | <0.001 | 48.54 (37.73 to 60.92) | 0.4 (0.19 to 0.61) | <0.001 |

**Abbreviation:** APC: annual percent change; ASDALYs: age-standardized disability-adjusted life years; ASDR: age-standardized death rate; ASIR: age-standardized incidence rate; CI: confidence interval: UI: uncertainty interval
